# An ICF-based content analysis of the overlap between questionnaires assessing tinnitus distress and depressive symptoms

**DOI:** 10.1371/journal.pone.0358644

**Published:** 2026-09-21

**Authors:** Denise Fuchten, Kelly K. S. Assouly, Inge Stegeman, Adriana L. Smit

**Affiliations:** 1 Department of Otorhinolaryngology, Head and Neck Surgery, University Medical Center Utrecht, Utrecht, The Netherlands; 2 University Medical Center Utrecht Brain Center, University Medical Center Utrecht, Utrecht, The Netherlands; 3 Cochlear Technology Centre, Mechelen, Belgium; University of Belgrade: Univerzitet u Beogradu, SERBIA

## Abstract

**Introduction:**

The experience of tinnitus varies considerably among individuals, particularly in terms of perceived distress. Psychological factors play a major role in this variability, and numerous studies have demonstrated a correlation between symptoms of depression and tinnitus distress. Both constructs are commonly assessed through self-report questionnaires. However, content overlap between these questionnaires can make it challenging to differentiate the constructs and interpret their relationship. Given the variation in content among questionnaires assessing tinnitus distress and depressive symptoms, a comprehensive examination of their similarities and differences is needed. This study therefore aims to systematically assess the content overlap of tinnitus distress and depressive symptom questionnaires using the International Classification of Functioning, Disability and Health (ICF) framework.

**Methods and analysis:**

Six validated, multi-item, self-report questionnaires measuring tinnitus distress (THI, TQ, mTQ, THQ, TRQ, TFI) and seven validated, multi-item, self-report depressive symptom questionnaires (BDI-II, HADS-D, SDS, PHQ-9, CES-D, SCL-90-R depression subscale, DASS-42 depression subscale) were included in the content analysis. The underlying concepts of all items of these questionnaires were linked to the most specific ICF categories based on established linking rules. The overlap between the tinnitus distress and depressive symptom questionnaires was analyzed based on the assigned second-level ICF categories.

**Results:**

The depressive symptom questionnaires demonstrated less diversity in ICF category coverage compared to the tinnitus distress questionnaires, with 14 versus 23 second-level ICF categories. Seven second-level categories were shared between tinnitus distress and depressive symptom questionnaires; energy and drive functions, sleep functions, attention functions, emotional functions, thought functions, interpersonal interactions and relationships, and recreation and leisure. The content analysis showed that, at the second-level ICF category, the TQ had the lowest degree of overlap with the depressive symptom questionnaires (11.54–26.92%), while the TRQ exhibited the highest (61.54–73.08%). Among the depressive symptom questionnaires, the SDS showed the least overlap with the tinnitus distress questionnaires (40–60%), whereas the DASS-42 depression subscale demonstrated the most overlap (85.71–100%).

**Conclusion:**

The overlap between tinnitus distress and depressive symptom questionnaires emphasizes the importance of carefully selecting assessment tools and interpreting their results, based on specific clinical or research goals. This content analysis can guide making decisions about this selection.

## Introduction

Tinnitus, the perception of sound in the absence of an external auditory stimulus [1], is a prevalent phenomenon estimated to affect around 740 million people worldwide [2]. While many people experience tinnitus, it is important to distinguish between its presence and its potential impact on individuals, as the majority of those affected do not experience significant distress from this phantom sound [2,3]. To address this distinction, de Ridder et al. proposed two separate terms [4]. They defined tinnitus as “the conscious awareness of a tonal or composite noise for which there is no identifiable corresponding external sound source”, while tinnitus disorder occurs when tinnitus is “associated with emotional and/or cognitive dysfunction, and/or autonomic arousal, leading to behavioral changes and functional disability” [4].

Although it is not yet fully understood why some people are severely impacted by tinnitus while others are less affected, research indicates that psychological factors play a major role in the variability of this perceived severity [5,6]. Among these psychological factors, depression is frequently found to be associated with the distress experienced by individuals with tinnitus [6–9]. Several hypotheses have been proposed to explain the link between tinnitus and depression. Some theories suggest that the impact of tinnitus can trigger depression in individuals who are prone to it, or that depression might cause a heightened focus on existing tinnitus, causing it to be perceived as more severe [10]. Other theories propose a more bidirectional relationship, shared neurobiological mechanisms, or common risk factors between tinnitus and depression [11]. Though the exact mechanism remains a topic of debate [8], the association between tinnitus and depression has consistently been observed. A systematic review by Meijers et al. (2022), which examined the relationship between tinnitus distress and depressive symptoms in observational studies, found significant positive correlations in 31 out of 33 included studies [9].

Studies examining this relationship often utilize self-report questionnaires for both tinnitus and depression assessment [11]. However, the content of these questionnaires partially overlaps, which can potentially lead to an overestimation of the correlation between depression and tinnitus [12]. For example, a study by Ooms et al. (2011) found content overlap in 15 out of 25 questions of the Tinnitus Handicap Inventory (THI) with 13 out of 21 questions of the Beck Depression Inventory (BDI) [12]. This overlap can complicate the differentiation of the two conditions and interpretation of their relationship, and may also impact treatment decisions when prioritizing interventions to reduce symptoms in clinical practice. Moreover, the overlap also poses challenges in participant selection in clinical trials. High scores on tinnitus distress measures may reflect depressive symptoms due to shared content, making it difficult to include patients who experience significant tinnitus burden but low depression levels [13]. This may inadvertently lead to the exclusion of eligible subjects, compromising the representativeness of study populations and limiting the generalizability of research findings.

Understanding the overlap requires considering what these questionnaires actually measure. Tinnitus questionnaires mainly operationalize tinnitus-related distress and impact, meaning the emotional, cognitive, and functional consequences of hearing the tinnitus sound in daily life, rather than the tinnitus percept, which refers solely to the auditory sensation itself. Depression questionnaires, in turn, measure depressive symptoms rather than providing a clinical diagnosis of depression. Part of the content overlap between these questionnaires can be attributed to shared symptomatology: patients with tinnitus often report problems with sleeping, difficulty concentrating, social withdrawal, and despair, symptoms that are also indicative of depression [11]. Both tinnitus distress and depression are heterogeneous, multidimensional constructs. Depression manifests as a set of partially overlapping and sometimes opposing symptom dimensions, including somatic, cognitive, affective, and behavioral dimensions, with questionnaires differing substantially in how they weight these dimensions [14]. Similarly, tinnitus distress can manifest across different domains, with questionnaires differing in their focus. For example, the Tinnitus Reaction Questionnaire (TRQ) is designed to evaluate the psychological distress associated with tinnitus [15] and therefore places greater emphasis on emotional functions compared to other tinnitus measures [16]. Given this heterogeneity, and the fact that different instruments operationalize these constructs by placing varying emphasis on specific symptom dimensions, overlap between questionnaires should be interpreted primarily as overlap between specific measurement instruments and their item content, rather than as direct evidence of overlap between the underlying constructs.

The variability in questionnaire focus underscores the need for a comprehensive examination of multiple tinnitus distress and depressive symptom questionnaires to better understand the nature and extent of their overlap. The current study aims to examine the overlap between tinnitus distress and depressive symptom questionnaires by analyzing their content based on the International Classification of Functioning, Disability and Health (ICF) framework. By using this standardized and internationally recognized framework to guide the content analysis, this study ensures a systematic examination of the items in the questionnaires. This analysis will provide a comprehensive understanding of the similarities and differences between the measures, and can guide future decisions regarding the selection of measurement tools.

## Methods

The methods of this study are detailed in a previously published protocol [17]. For a more comprehensive description of the methodology and questionnaires used, readers are advised to consult the published protocol of our study. No ethical approval was required for this study, due to the characteristics of the study design.

### Questionnaires

The content analysis included a selection of validated, multi-item, self-report questionnaires for assessing tinnitus distress and depressive symptoms. The included tinnitus distress questionnaires are the Tinnitus Handicap Inventory (THI), Tinnitus Questionnaire (TQ), mini Tinnitus Questionnaire (mTQ), Tinnitus Handicap Questionnaire (THQ), Tinnitus Reaction Questionnaire (TRQ) and Tinnitus Functional Index (TFI) [15,18–23]. These questionnaires have been widely utilized in both clinical practice and research in order to assess the severity of symptoms, and are recommended or referenced in multiple international clinical practice guidelines [24–31]. The included depressive symptom questionnaires are the Beck Depression Inventory-II (BDI-II), Hospital Anxiety and Depression Scale (HADS) depression subscale, Zung Self-Rating Depression scale (SDS), Patient Health Questionnaire-9 (PHQ-9), Center for Epidemiologic Studies Depression Scale (CES-D), Symptom Checklist-90-Revised (SCL-90-R) depression subscale and Depression Anxiety Stress Scale (DASS) depression subscale [32–38]. These questionnaires are commonly used in research on tinnitus based on two systematic reviews on tinnitus and depression [8,9].

### ICF linking procedure

#### ICF linking rules.

**Fig 1:**
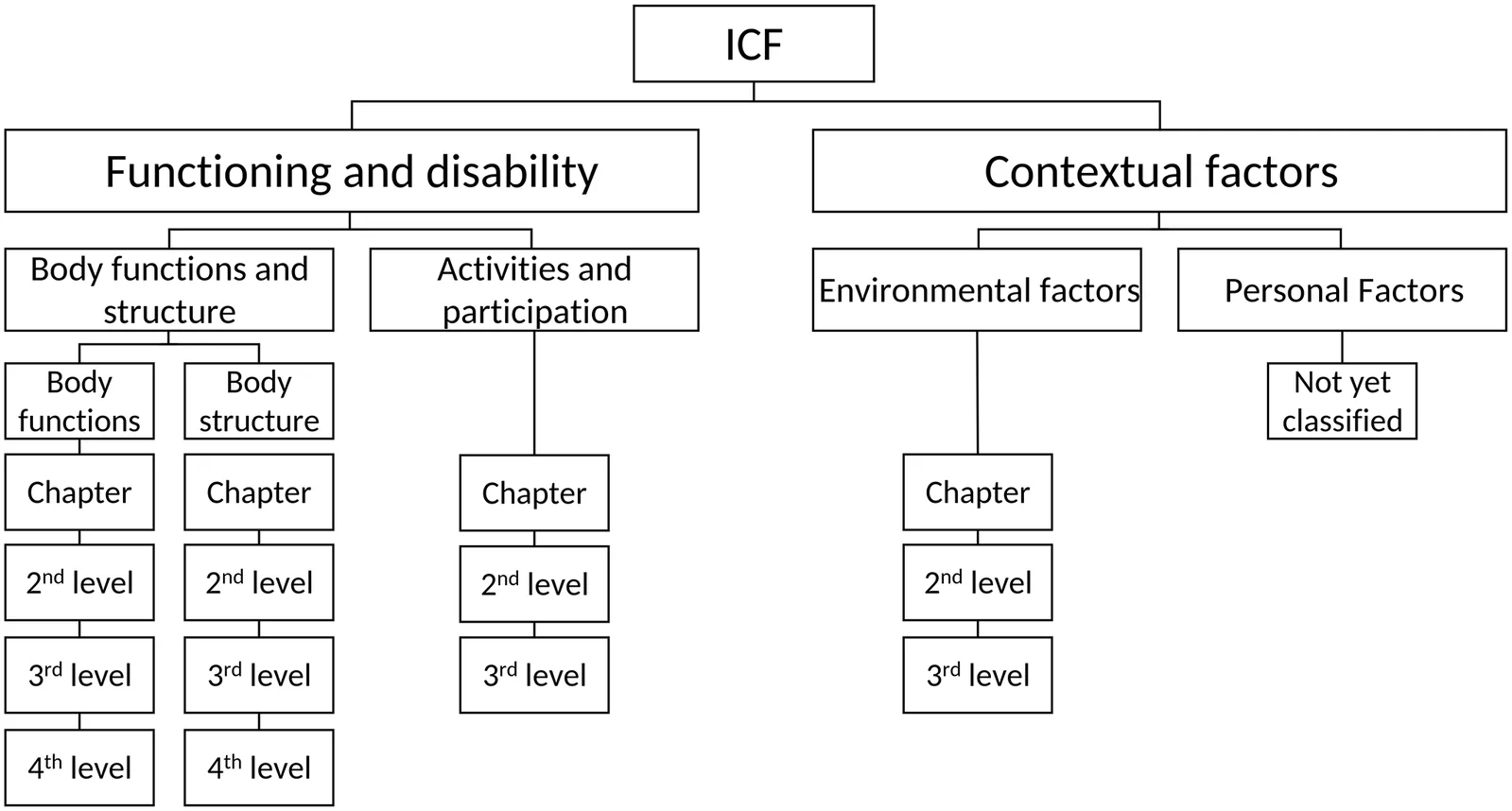
Hierarchical structure of the ICF Framework.

The International Classification of Functioning, Disability and Health (ICF) framework, developed by the World Health Organization, was used to analyze the content of the tinnitus distress and depressive symptom questionnaires [39]. As illustrated in Fig 1, the ICF is organized into two main parts; (1) functioning and disability, which includes the components body functions and structures as well as activities and participation, and (2) contextual factors, consisting of environmental and personal factors. With the exception of personal factors, each component is further categorized in levels with corresponding codes.

Each questionnaire item was linked to the ICF framework based on the linking rules developed and refined by Cieza et al [40–42]. In accordance with these rules, the main concept and, when applicable, additional concepts of each item of the questionnaires were identified, after which they were linked to the most precise ICF category. An item could only be assigned to more than one category when it contained multiple concepts. For concepts not adequately represented in the ICF framework, specific codes were assigned: ‘nc’ (not covered) for concepts absent from the ICF, ‘pf’ for personal factors not further classified in the ICF framework, and ‘nd’ (not definable) when item information was insufficient for precise ICF category linking. Further elaboration on the most recent version of the linking rules can be found in the article of Cieza et al. published in 2019 [42]. However, two steps outlined in this article, identification of the perspective and identification of the response options, were not applied in the current study. As the perspective from which an item was written and the type of response scale used do not affect what concept an item measures, these rules were not relevant for the purpose of the current analysis. Table 1 shows an example of the linking of a questionnaire item to the ICF framework.

**Table 1:** Example of linking a questionnaire item (*TFI item 2. Over the past week, how strong or loud was your tinnitus?*) to the hierarchical structure of the ICF Framework.

Level	Example	Coding
Component	Body functions	b
Chapter (first level)	Sensory functions and pain	b2
Second level	Sensations associated with hearing and vestibular function	b240
Third level	Ringing in ears or tinnitus	b2400

#### Consensus procedure and additional coding decisions.

All items were linked by two independent reviewers (DF and KKSA), a psychologist and a biomedical researcher. Upon comparing their initial categorizations, some differences in the interpretation of specific ICF category codes were identified, which prompted discussion and revision of the coding of ambiguous or discrepant items across the questionnaires. This process of coding, comparison, and discussion was iterative in nature.

Several interpretive decisions were made after discussion between the two reviewers, either to resolve different initial interpretations, or to address items whose content was inherently more complex to map onto the ICF framework. First, items in which tinnitus itself was the direct object of the concept being measured were treated as inseparable from the tinnitus experience, and were therefore linked to *sensations associated with* *hearing and vestibular function (b240)*, and more specifically to the third-level category *ringing in ears or tinnitus (b2400)*. For example, the item “Over the past week, how easy was it for you to ignore your tinnitus?” was categorized under this code because the concept being measured is ‘ignoring’, with tinnitus as its direct object. In contrast, the item “Over the past week, how much did your tinnitus interfere with your ability to concentrate?” was not coded in this manner, as the concept being measured is concentration, with tinnitus representing the source of the difficulty rather than its direct object. This item was therefore linked to *attention functions (b140)*. This approach was chosen to provide a consistent method for coding tinnitus-specific items when comparing tinnitus distress questionnaires with depressive symptom measures. The effect of this specific coding decision on the reported overlap was subsequently examined using an alternative coding strategy, described under ‘Alternative coding of b240 items’.

Second, for items referring to hearing and listening contexts a distinction was made following Granberg et al. (2014) [43]. Hearing as a passive function was linked to *hearing functions (b230)*, whereas listening as an active and intentional process was linked to *listening (d115).* Items involving listening with comprehension were linked to *communicating with – receiving – spoken messages (d310),* and items involving bidirectional communication were linked to *interpersonal interactions and relationships (d799).*

Third, the category *temperament and personality functions (b126),* defined as ‘general mental functions of constitutional disposition of the individual to react in a particular way to situations, including the set of mental characteristics that makes the individual distinct from others’, was not applied, as the definition relates to personality traits rather than psychological states [44], and the items in the questionnaires assess a change rather than a constant.

Following this iterative process, a third reviewer (ALS), an ENT specialist as well as an epidemiologist, was consulted to reach consensus on items that remained unresolved after discussion between the two primary reviewers. These generally concerned two types of issues: individual items where the appropriate ICF category was unclear (e.g., whether certain hearing-related items involved comprehension) and groups of conceptually similar items across questionnaires for which a consistent coding rule was established. In addition to unresolved items, discussions with the third reviewer also included a small number of items on which the two primary reviewers had already agreed, but which were re-examined as a verification check.

Several recurring themes were identified during the sessions with the third reviewer for which consistent coding decisions were made. Items related to enjoyment of life, such as life feeling full, meaningless, or not worthwhile, were coded as *nc-qol*. Items reflecting negative self-perception, such as feelings of insecurity, loss of confidence, or feeling like a failure, were linked to *emotional functions (b152),* as the third-level category *confidence (b1266)* under temperament and personality functions was not applied in the current study. Similarly, items regarding feeling hopeless, helpless or discouraged were also linked to *emotional functions (b152).*

#### Alternative coding of b240 items.

The coding decision of the *b240* (*hearing and vestibular function*) items, as described above, represents a deliberate choice made for the purpose of comparing tinnitus distress and depressive symptom questionnaires. For these items, tinnitus is named as the direct object of the concept being measured (e.g., coping with tinnitus, ignoring tinnitus), and this decision means that the concept is treated as inseparable from the tinnitus experience itself. However, an alternative interpretation is possible for these items: what is measured may instead be a more general process (e.g., a general capacity for coping), which manifests here in relation to tinnitus but might not necessarily be specific to it. To assess the extent to which this alternative interpretation affects the reported overlap, an alternative coding strategy was applied in which such items were recoded, where a plausible ICF category could be identified for the underlying process in question. Items whose content could only be understood as describing the tinnitus percept itself, such as its loudness or presence, remained coded under *b240*.

### Data analysis

Content analysis was performed on the second-level codes assigned to each item of the included questionnaires. Frequencies of the assigned second-level codes per questionnaire were reported as absolute numbers and percentages. Additionally, frequencies of the assigned second-level codes for all tinnitus distress questionnaires combined and for all depressive symptom questionnaires combined were reported as absolute numbers and percentages. Since the mTQ is an abridged version of the TQ and therefore does not contain unique items, it was excluded from the combined reporting of second-level code frequencies.

Subsequently, comparisons were made between each tinnitus distress questionnaire and each depressive symptom questionnaire to assess item overlap, defined as the number of items linked to the same second-level ICF code across the two questionnaires. This overlap was reported both as absolute numbers and percentages.

Using the alternative coding described in the ICF linking procedure, overlap between the tinnitus distress and depressive symptom questionnaires was recalculated following the same procedure as in the main analysis. This sensitivity analysis was supplementary to the main analysis; the original *b240* coding was retained throughout the main analysis, and all overlap results reported in the results, discussion, and conclusion refer to the main analysis unless stated otherwise.

Agreement between the two primary reviewers on the second-level ICF codes assigned during the main coding process was calculated as a percentage agreement, prior to discussion and consensus. For items where a reviewer had assigned multiple candidate codes, to be resolved through discussion with the other reviewer, agreement was defined as at least one of these codes matching the code assigned by the other reviewer. For this reason, a standard kappa coefficient was not deemed a suitable measure of agreement for this dataset, and inter-rater agreement was instead reported as a percentage agreement, in contrast to what was outlined in the study protocol [17].

## Results

### Inter-rater agreement

Initial agreement between the two reviewers on the second-level ICF codes was 75.67% (199 of 263 items). Following discussion between the two reviewers and the establishment of additional coding rules, 76 items (29.01% of all items) were discussed with the third reviewer, including a number of items that had already been agreed upon but were checked for verification. Several of these 76 items were conceptually similar and recurred across different questionnaires, for example items reflecting negative self-perception, such as feelings of insecurity, loss of confidence, or feeling like a failure. While such recurring items were discussed as a group, each was still counted as a separate item within the total of 76.

### Categorization of tinnitus distress questionnaire items

Table 2 shows the categorization of concepts across the tinnitus distress questionnaire items. The total number of items across the five questionnaires (THI, TQ, THQ, TRQ, TFI) is 155, classified into 162 second-level ICF codes. The higher number of codes compared to the number of items is due to two items in both the TFI and the THI containing multiple concepts. For example, item 13 of the THI “Does your tinnitus interfere with your job or household responsibilities?” includes both the concepts ‘interference with job’ and ‘interference with household responsibilities’, resulting in multiple categorizations.

**Table 2:** Categorization of concepts within the tinnitus distress questionnaire items.

	THI (k = 25)	TQ (k = 52)	THQ (k = 27)	TRQ (k = 26)	TFI (k = 25)	Total (k = 155)	mTQ (k = 12)
**Body functions**	**19 (70.37%)**	**40 (76.92%)**	**13 (48.15%)**	**18 (69.23%)**	**16 (53.33%)**	**106 (65.43%)**	**8 (66.67%)**
b130 Energy and drive functions	1 (3.70%)	–	1 (3.70%)	–	–	2 (1.23%)	–
b134 Sleep functions	1 (3.70%)	5 (9.62%)	1 (3.70%)	1 (3.85%)	3 (10.00%)	11 (6.79%)	1 (8.33%)
b140 Attention functions	1 (3.70%)	2 (3.85%)	1 (3.70%)	1 (3.85%)	1 (3.33%)	6 (3.70%)	1 (8.33%)
b152 Emotional functions	8 (29.63%)	5 (9.62%)	6 (22.22%)	15 (57.69%)	3 (10.00%)	37 (22.94%)	2 (16.67%)
b160 Thought functions	1 (3.70%)	–	–	1 (3.85%)	1 (3.33%)	3 (1.85%)	–
b230 Hearing functions	1 (3.70%)	2 (3.85%)	1 (3.70%)	–	1 (3.33%)	5 (3.09%)	–
b240 Sensations associated with hearing and vestibular function	6 (22.22%)	23 (44.23%)	3 (11.11%)	–	7 (23.33%)	39 (24.07%)	4 (33.33%)
b280 Sensation of pain	–	2 (3.85%)	–	–	–	2 (1.23%)	–
b735 Muscle tone functions	–	1 (1.92%)	–	–	–	1 (0.62%)	–
**Activities and participation**	**6 (22.22%)**	**6 (11.54%)**	**8 (29.63%)**	**4 (15.38%)**	**12 (40.00%)**	**36 (22.22%)**	**1 (8.33%)**
d115 Listening	–	2 (3.85%)	3 (11.11%)	–	1 (3.33%)	6 (3.70%)	–
d166 Reading	1 (3.70%)	–	–	–	–	1 (0.62%)	–
d240 Handling stress and other psychological demands	–	–	1 (3.70%)	–	–	1 (0.62%)	–
d310 Communicating with – receiving – spoken messages	–	1 (1.92%)	1 (3.70%)	–	1 (3.33%)	3 (1.85%)	–
d649 Household tasks other specified and unspecified	1 (3.70%)	–	–	–	1 (3.33%)	2 (1.23%)	–
d699 Domestic life, unspecified	–	–	–	–	1 (3.33%)	1 (0.62%)	–
d750 Informal social relationship	1 (3.70%)	–	–	–	1 (3.33%)	2 (1.23%)	–
d760 Family relationships	1 (3.70%)	–	1 (3.70%)	–	1 (3.33%)	3 (1.85%)	–
d799 Interpersonal interactions and relationships, unspecified	–	–	1 (3.70%)	–	1 (3.33%)	2 (1.23%)	–
d839 Education, other specified and unspecified	–	–	–	–	1 (3.33%)	1 (0.62%)	–
d859 Work and employment other specified and unspecified	1 (3.70%)	–	–	1 (3.85%)	1 (3.33%)	3 (1.85%)	–
d920 Recreation and leisure	1 (3.70%)	3 (5.77%)	1 (3.70%)	3 (11.54%)	3 (10.00%)	11 (6.79%)	1 (8.33%)
**Environmental factors**	**–**	**1 (1.92%)**	**2 (7.40%)**	**–**	**–**	**3 (1.85%)**	**–**
e320 Friends e398 Other specified support and relationships e399 Support and relationships, unspecified	– – –	– – 1 (1.92%)	1 (3.70%) 1 (3.70%) –	– – –	– – –	1 (0.62%) 1 (0.62%) 1 (0.62%)	– – –
**Not covered/defined**	**2 (7.41%)**	**5 (9.62%)**	**4 (14.81%)**	**4 (15.38%)**	**2 (6.67%)**	**17 (10.49%)**	**3 (25.00%)**
Not covered	1 (3.70%)	3 (5.77%)	3 (11.11%)	3 (11.54%)	1 (3.33%)	11(6.79%)	2 (16.67%)
Not covered – quality of life	1 (3.70%)	2 (3.85%)	1 (3.70%)	1 (3.85%)	1 (3.33%)	6 (3.70%)	1 (8.33%)
**Total 2**^**nd**^ **level categories**	**27**	**52**	**27**	**26**	**30**	**162**	**12**

*THI*, Tinnitus Handicap Inventory; *TQ*, Tinnitus Questionnaire; *mTQ*, mini Tinnitus Questionnaire; *THQ*, Tinnitus Handicap Questionnaire; *TRQ*, Tinnitus Reaction Questionnaire; *TFI*, Tinnitus Functional Index.The mTQ is presented after the total column and is not included in the total counts and percentages, as it is an abridged version of the TQ and therefore does not contain unique items.

The majority of concepts within the tinnitus distress questionnaires belong to the component *body functions*, accounting for 106 codes (65.43%). Within this component, the second-level code *b240 sensations associated with hearing and vestibular function* is most prominently represented, as concepts were assigned to this level 39 times (24.07%). All of these instances were linked to the third-level code *b2400 ringing in ears or tinnitus.* The second most prominent second-level code within the component *body functions* is *b152 emotional functions*, with 37 concepts (22.94%).

The component *activities and participation* consists of the most items after *body functions*, with a total of 36 concepts (22.22%) assigned to this component. Most concepts were related to leisure activities, with 11 concepts (6.79%) linked to the code *d920 recreation and leisure*. Following this, items reflecting difficulties in listening contexts, both without (*d115 listening*) and with (*d310 communicating with – receiving – spoken messages)* the necessity of comprehension, were represented, respectively 6 (3.7%) and 3 (1.85%) times.

Three item across all tinnitus distress questionnaires had a main concept related to the component *environmental factors*, all most closely corresponding to the chapter *support and relationships*.

In total, 17 (10.49%) concepts of the tinnitus distress questionnaires could not be linked to an existing ICF category and were therefore coded *not covered*. Of these, 6 (3.7%) encompassed enjoyment of life, and where therefore coded *nc-qol (not covered – quality of life*). The remaining 11 (6.79%) concepts included themes such as health concerns, avoiding certain sound environments, complaining and suicide.

The coding of all tinnitus distress questionnaire items can be found in the supporting material (S1 File).

### Categorization of depressive symptom questionnaire items

Table 3 shows the categorization of concepts across the depressive symptom questionnaire items. The total number of items across all six questionnaires is 107, categorized into an equal amount of second-level ICF codes.

**Table 3:** Categorization of concepts within the depressive symptom questionnaire items.

	BDI-II (k = 21)	HADS-D (k = 7)	SDS (k = 20)	PHQ-9 (k = 9)	CES-D (k = 20)	SCL-90-R (k = 16)	DASS-42 (k = 14)	Total (k = 107)
**Body functions**	**17 (80.95%)**	**3 (42.86%)**	**15 (75.00%)**	**7 (77.79%)**	**15 (75.00%)**	**13 (81.25%)**	**8 (57.14%)**	**78 (72.90%)**
b130 Energy and drive functions	3 (14.29%)	1 (14.29%)	3 (15.00%)	2 (22.22%)	3 (15.00%)	2 (12.50%)	2 (14.29%)	16 (14.95%)
b134 Sleep functions	1 (4.76%)	–	1 (5.00%)	1 (11.11%)	1 (5.00%)	–	–	4 (3.74%)
b140 Attention functions	1 (4.76%)	–	–	1 (11.11%)	1 (5.00%)	–	–	3 (2.80%)
b147 Psychomotor functions	1 (4.76%)	–	1 (5.00%)	1 (11.11%)	–	–	–	3 (2.80%)
b152 Emotional functions	10 (47.62%)	2 (28.57%)	5 (25.00%)	2 (22.22%)	10 (50.00%)	8 (50.00%)	6 (42.86%)	43 (40.19%)
b160 Thought functions	–	–	1 (5.00%)	–	–	2 (12.50%)	–	3 (2.80%)
b410 Heart functions	–	–	1 (5.00%)	–	–	–	–	1 (0.93%)
b530 Weight maintaining functions	–	–	1 (5.00%)	–	–	–	–	1 (0.93%)
b525 Defecation functions	–	–	1 (5.00%)	–	–	–	–	1 (0.93%)
b640 Sexual functions	1 (4.76%)	–	1 (5.00%)	–	–	1 (6.25%)	–	3 (2.80%)
**Activities and participation**	**1 (4.76%)**	**1 (14.29%)**	**1 (5.00%)**	**–**	**3 (15.00%)**	**–**	**–**	**6 (5.61%)**
d177 Making decisions	1 (4.76%)	–	1 (5.00%)	–	–	–	–	2 (1.87%)
d330 Speaking	–	–	–	–	1 (5.00%)	–	–	1 (0.93%)
d799 Interpersonal interactions and relationships, unspecified	–	–	–	–	2 (10.00%)	–	–	2 (1.87%)
d920 Recreation and leisure	–	1 (14.29%)	–	–	–	–	–	1 (0.93%)
**Not covered/defined**	**3 (14.29%)**	**3 (42.86%)**	**4 (20.00%)**	**2 (22.22%)**	**2 (10.00%)**	**3 (18.75%)**	**6 (42.86%)**	**23 (21.50%)**
Not covered	1 (4.76%)	1 (14.29%)	1 (5.00%)	1 (11.11%)	–	2 (12.50%)	–	6 (5.61%)
Not covered – quality of life	2 (9.52%)	2 (28.57%)	2 (10.00%)	1 (11.11%)	2 (10.00%)	1 (6.25%)	6 (42.86%)	16 (14.95%)
Not defined	–	–	1 (5%)	–	–	–	–	1 (0.93%)
**Total 2**^**nd**^ **level categories**	**21**	**7**	**20**	**9**	**20**	**16**	**14**	**107**

*BDI-II*, Beck Depression Inventory-II; *HADS-D*, Hospital Anxiety and Depression Scale – depression subscale; *SDS*, Zung Self-Rating Depression Scale; *PHQ-9*, Patient Health Questionnaire-9; *CES-D*; Center for Epidemiologic Studies Depression Scale; *SCL-90-R*, Symptom Checklist-90-Revised; *DASS-42*, Depression Anxiety Stress Scale 42

As with the tinnitus distress questionnaires, the majority of concepts within the depressive symptom questionnaires belong to the component *body functions*, accounting for 78 codes (72.90%). Within this component, the second-level code *b152 emotional functions* is most prominently represented with 43 items (40.19%). *B130 energy and drive functions* is the second most prominent category, with 16 items (14.95%) regarding energy level, motivation, appetite or other specified energy and drive functions.

Six items (5.61%) were attributed to the component *activities and participation*. This encompassed items regarding making decisions, interpersonal interactions and relationships, recreation and leisure, and speaking.

In total, 23 items (21.50%) could not be linked to an existing ICF category. Of these, 16 (14.95%) were coded *nc-qol* and involved concepts regarding loss of enjoyment of life or interest in things. The other 6 items (5.61%) mainly concerned suicidal thoughts and were coded *nc.* One item (0.95%) could not be defined (SDS item 2 “Morning is when I feel best”) and therefore got assigned the code *nd*.

The coding of all depressive symptom questionnaire items can be found in the supporting material (S2 File).

### Overlap between tinnitus distress and depressive symptom questionnaires

**Fig 2:**
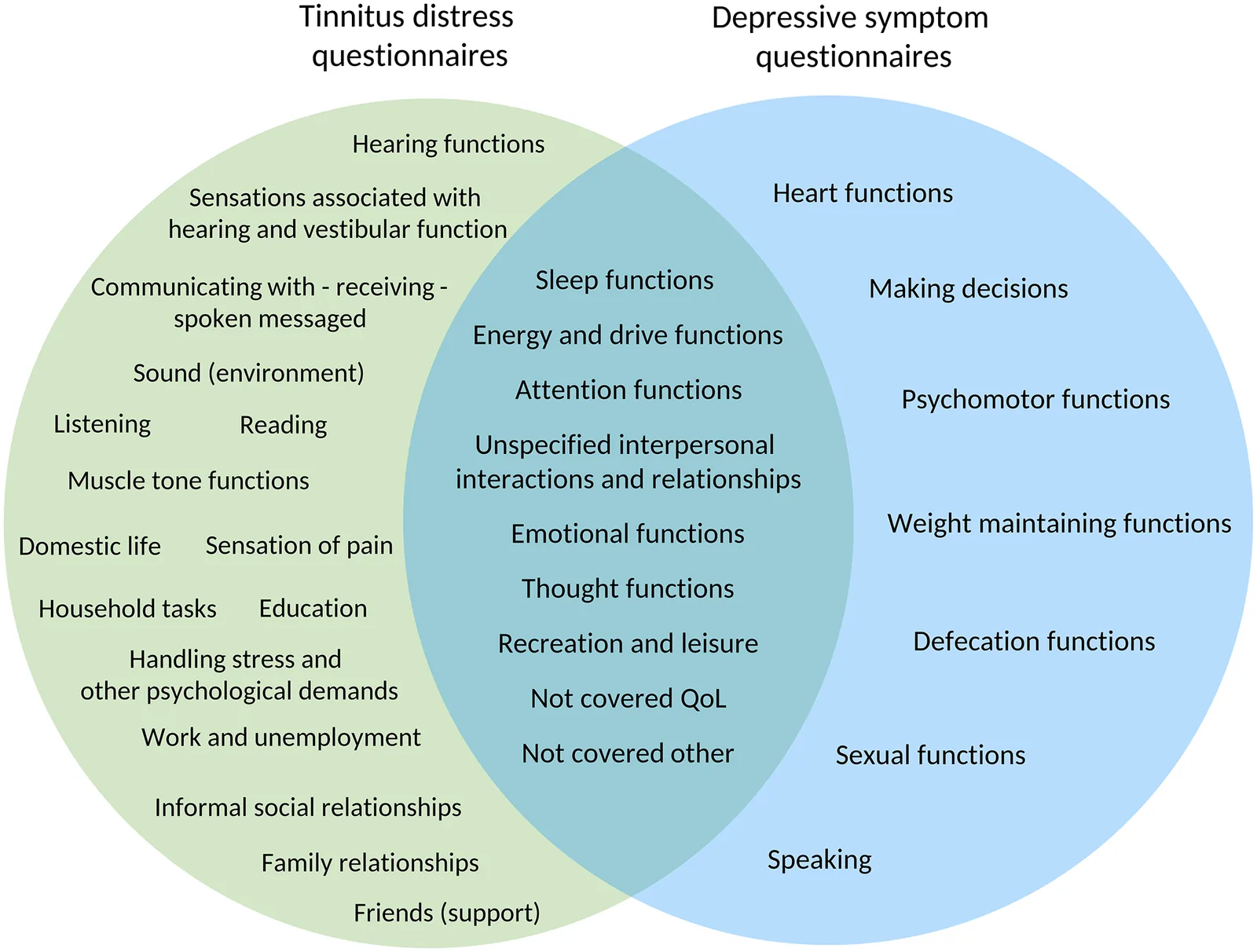
Venn diagram of second-level ICF-categories in tinnitus distress and depressive symptom questionnaires.

When comparing the second-level categories of the tinnitus distress and depressive symptom questionnaires, seven categories are shared between both sets of questionnaires; *b130 energy and drive functions, b134 sleep functions, b140 attention functions, b152 emotional functions, b160 thought functions, d799 interpersonal interactions and relationships, unspecified,* and *d920 recreation and leisure*. In addition to these overlapping categories, both the tinnitus distress and depressive symptom questionnaires also contained items that could not be linked to any ICF category. Fig 2 illustrates the second-level categories found in both types of questionnaires and highlights the areas of overlap.

Cross-tables comparing each tinnitus distress questionnaire with each depressive symptom questionnaire, provided in the supporting material (S3 File), detail the specific items that overlap in terms of ICF categorization. For this comparison, the category *nc-qol* was also included, as it pertains specifically to items related to enjoyment of life, making it directly comparable across the questionnaires. However, the broader *nc* category was excluded from the comparison due to its diverse range of items that do not lend themselves to clear overlap.

Table 4 illustrates the extent of overlap in ICF-categories between the items of different tinnitus distress and depressive symptom questionnaires. For instance, it highlights how 12 out of 25 items (48%) from the THI overlap with 17 of the 21 items (80.95%) from the BDI based on the assigned second-level codes of the ICF framework.

**Table 4:** Amount of item overlap between tinnitus distress questionnaires and depressive symptom questionnaires based on second-level ICF categorization.

	BDI-II		HADS-D		SDS		PHQ-9		CES-D		SCL-90-R		DASS-42	
**THI**	THI	BDI-II	THI	HADS-D	THI	SDS	THI	PHQ-9	THI	CES-D	THI	SCL-90	THI	DASS-42
	12/25 48.00%	17/21 80.95%	11/25 44.00%	6/7 85.71%	12/25 48.00%	12/20 60.00%	12/25 48.00%	7/9 77.78%	12/25 48.00%	17/20 85.00%	11/25 44.00%	13/16 81.25%	10/25 40.00%	14/14 100.00%
**TQ**	TQ	BDI-II	TQ	HADS-D	TQ	SDS	TQ	PHQ-9	TQ	CES-D	TQ	SCL-90	TQ	DASS-42
	14/52 26.92%	14/21 66.67%	10/52 19.23%	5/7 71.43%	12/52 23.08%	8/20 40.00%	14/52 26.92%	5/9 55.56%	14/52 26.92%	14/20 70.00%	7/52 13.46%	9/16 56.25%	6/52 11.54%	12/14 85.71%
**mTQ**	mTQ	BDI-II	mTQ	HADS-D	mTQ	SDS	mTQ	PHQ-9	mTQ	CES-D	mTQ	SCL-90	mTQ	DASS-42
	5/12 41.67%	14/21 66.67%	4/12 33.33%	5/7 71.43%	4/12 33.33%	8/20 40.00%	5/12 41.67%	5/9 55.56%	5/12 41.67%	14/20 70.00%	3/12 25.00%	9/16 56.25%	3/12 25.00%	12/14 85.71%
**THQ**	THQ	BDI-II	THQ	HADS-D	THQ	SDS	THQ	PHQ-9	THQ	CES-D	THQ	SCL-90	THQ	DASS-42
	10/27 37.04%	17/21 80.95%	9/27 33.33%	6/7 85.71%	9/27 33.33%	11/20 55.00%	10/27 37.04%	7/9 77.78%	11/27 40.74%	19/20 95.00%	8/27 29.63%	11/16 68.75%	8/27 29.63%	14/14 100.00%
**TRQ**	TRQ	BDI-II	TRQ	HADS-D	TRQ	SDS	TRQ	PHQ-9	TRQ	CES-D	TRQ	SCL-90	TRQ	DASS-42
	18/26 69.23%	14/21 66.67%	19/26 73.08%	5/7 71.43%	18/26 69.23%	9/20 45.00%	18/26 69.23%	5/9 55.56%	18/26 69.23%	14/20 70.00%	17/26 65.38%	11/16 68.75%	16/26 61.54%	12/14 85.71%
**TFI**	TFI	BDI-II	TFI	HADS-D	TFI	SDS	TFI	PHQ-9	TFI	CES-D	TFI	SCL-90	TFI	DASS-42
	8/25 32.00%	14/21 66.67%	7/25 28.00%	5/7 71.43%	8/25 32.00%	9/20 45.00%	8/25 32.00%	5/9 55.56%	9/25 36.00%	16/20 80.00%	5/25 20.00%	11/16 68.75%	4/25 16.00%	12/14 85.71%

*BDI-II*, Beck Depression Inventory-II; *HADS-D*, Hospital Anxiety and Depression Scale – depression subscale; *SDS*, Zung Self-Rating Depression Scale; *PHQ-9*, Patient Health Questionnaire-9; *CES-D*; Center for Epidemiologic Studies Depression Scale; *SCL-90-R*, Symptom Checklist-90-Revised; *DASS-42*, Depression Anxiety Stress Scale 42

This table presents the item overlap between various tinnitus distress and depressive symptom questionnaires based on second-level ICF categorization. For example, 12 out of 25 items (48%) from the THI overlap with the BDI, and 17 out of 21 items (80.95%) from the BDI-II overlap with the THI.

When looking at the tinnitus distress questionnaires, the content of the TQ exhibits the least amount of overlap with the different depressive symptom questionnaires, with overlap ranging from 11.54% to 26.92% of items, followed by the TFI, which shows overlap ranging from 16% to 36% of its items. The mTQ and THQ demonstrate similar ranges, 25% to 41.67% and 29.63% to 40.74% respectively. The THI items overlap between 40% and 48% with the depressive symptom, questionnaires, while the TRQ shows the highest amount of overlap, ranging from 61.54% to 73.08%.

When assessing this overlap from the perspective of the depressive symptom questionnaires, overall, these questionnaires exhibit higher percentages of overlap with the different tinnitus distress questionnaires. The SDS has the least amount of overlap with the tinnitus distress questionnaires, ranging from 40% to 60% of items. The PHQ-9 and SCL-90 show similar overlap ranges of 55.56% to 77.78% and 56.25% to 81.25%, respectively. The BDI-II overlaps between 66.67% and 80.95% with the tinnitus distress questionnaires, while the HADS-D shows an overlap of 71.43% to 85.17% and the CES-D of 70% to 95%. Lastly, the DASS-42 shows the highest amount overlap, with a range from 85.71% to 100%.

### Alternative coding of b240 items

The full results of the sensitivity analysis using the alternative coding can be found in the supporting material (S4 File). This recoding distributed the affected items across *attention functions (b140)* and *emotional functions (b152).* Items describing coping or control in relation to tinnitus could not be assigned to any ICF category and were coded *not covered*, while items describing the tinnitus percept itself remained coded under *b240*.

Under this alternative coding, the TQ’s overlap with the depressive symptom questionnaires increased to 21.15–42.31%, and the TFI’s overlap increased to 20.00–48.00%. The THQ’s overlap with the depressive symptom questionnaires remained unchanged at 29.63–40.74%. The mTQ’s overlap increased to 33.33–58.33%, and the THI’s overlap increased to 40.00–52.00%. The TRQ’s overlap with the depressive symptom questionnaires remained unchanged at 61.54–73.08%, continuing to show the highest degree of overlap.

## Discussion

In this study we examined the content overlap between tinnitus distress and depressive symptom questionnaires using the ICF framework. By analyzing the content of these questionnaires, we sought to provide a comprehensive overview of their similarities and differences at the level of questionnaire operationalization, which can guide future decisions regarding the selection of measurement tools in both research and clinical practice.

While individual tinnitus distress and depressive symptom questionnaires differ in their specific content, reflecting their intended scope and aim, both groups of questionnaires primarily focus on the ICF component *body functions*. Within the ICF framework, the component *body functions* not only encompasses physiological functions but also mental functions. Tinnitus distress questionnaires had 48.15–76.92% of items categorized under this component, and depressive symptom questionnaires 42.86–81.25%. This emphasis reflects the established main focus of tinnitus distress questionnaires on the sensory and broader mental dimensions of tinnitus, including the cognitive-emotional impact [16], and the traditional classification and conceptualization of depression, which primarily targets internal symptoms such as described in the Diagnostic and Statistical Manual of Mental Disorders 5th Edition (DSM-5) [45]. Within the *body functions* component, the second-level category *emotional functions* was represented in all included questionnaires. It was also the most prevalent category in the depressive symptom questionnaires, accounting for 40.19% of all items, and the second most prevalent in tinnitus distress questionnaires, comprising 22.94% of items. The second-level category *sensations associated with hearing and vestibular function*, consistently linked to the third-level category *ringing in ears or tinnitus*, however, was the most prevalent in the tinnitus distress questionnaires (24.07% of all items).

Moving beyond *body functions*, the ICF component *activities and participation* showed a different pattern across the two questionnaire types. While tinnitus distress questionnaires had 8.33–40% of their items linked to this component, with the second-level category *recreation and leisure* represented in all tinnitus questionnaires, depressive symptom questionnaires showed less coverage of this component (0–15% of items), again reflecting their primary focus on internal symptoms. Items categorized under the ICF component *environmental factors* were rare overall, appearing in only three items across tinnitus distress questionnaires and none in depressive symptom questionnaires. Finally, both questionnaire types included items that could not be linked to an ICF category and were coded as *not covered*, with *quality of life* being an uncategorized theme present in all included tinnitus distress and depressive symptom questionnaires.

Overall, the depressive symptom questionnaires demonstrated less diversity in ICF categories compared to the tinnitus distress questionnaires, with a total of 14 different second-level categories versus 23 in the tinnitus distress questionnaires. An examination of the overlap between the questionnaires on the second level, shows that both types of questionnaires contain *energy and drive functions, sleep functions, attention functions, emotional functions, thought functions, interpersonal interactions and relationships,* and *recreation and leisure*.

When looking at this second-level overlap from the perspective of the tinnitus distress questionnaires, overall the TQ exhibits the least amount of overlap with the different depressive symptom questionnaires, which may partly reflect the large amount of items attributed to *sensations associated with hearing and vestibular function*, followed by the TFI due to its extensive coverage of items within the component *activities and participation* which were not prevalent in the depressive symptom questionnaires. The TRQ overall shows the highest amount of overlap with the depressive symptom questionnaires as a result of the large number of items attributed to *emotional functions* within this questionnaire.

When assessing this second-level overlap from the perspective of the depressive symptom questionnaires, overall, these questionnaires exhibit higher percentages of overlap with the different tinnitus distress questionnaires. This is because the depressive symptom questionnaires have less diversity in second-level categorization, and contain a large number of items related to the category *emotional functions* which are also present in every tinnitus distress questionnaire. The DASS-42 shows the highest amount of overlap with the tinnitus distress questionnaires, as it covers only three categories, two of which, *emotional functions* and *not covered-quality of life*, are present in each tinnitus distress questionnaire. Due to the diversity of the SDS, which also includes a range of physiological items and items focusing on speaking and interpersonal interactions and relationships, this questionnaire shows the least amount of overlap with tinnitus distress questionnaires.

While the degree of overlap between different tinnitus distress and depressive symptom questionnaires varies, it is important to acknowledge that overall there is a substantial overlap between these questionnaires. This overlap can partially be explained by shared symptomology between tinnitus and depression. Patients with tinnitus often report complaints such as problems with sleeping, problems with concentration, social withdrawal and despair, symptoms that are also indicative of depression [11]. However, the overlap in questionnaires can complicate the differentiation of symptoms being related to tinnitus or having a depressive status or depression, and potentially lead to misinterpretation of questionnaire results. Furthermore, the significant overlap in questionnaire items may inflate correlations between tinnitus and depression scores, possibly resulting in an overestimation of their relationship [12]. Considering these factors, clinicians and researchers should carefully interpret questionnaire results, taking into account the potential influence of overlapping content on scores. As questionnaire results can affect treatment decisions and prioritization of interventions in clinical settings, and can inform participant selection in clinical trials, careful selection of questionnaires aligned with specific goals is essential. For instance, if the aim of a clinical trial is to distinguish between tinnitus distress and depression symptom scores, the use of highly overlapping combinations such as the TRQ and DASS-42 might be avoided. Researchers and clinicians should also consider their target population and reflect on whether overlap in symptomatology captured by questionnaires is problematic or rather inherent to the population. In clinical trials, for example, depressive symptom questionnaires are often used to exclude participants with depressive symptoms that may interfere with treatment outcomes. In a recent clinical trial assessing a neuromodulation technique for tinnitus, almost all participants experiencing severe tinnitus distress also had elevated scores on a depression and anxiety questionnaire and therefore did not meet the study criteria [13]. Consequently, the study amended its in- and exclusion criteria to better align with the characteristics of the target population and to allow for more representative recruitment, as these psychological complaints might be inherent to the population with severe tinnitus [13]. This highlights the importance of carefully considering both questionnaire content and the characteristics of the target population when selecting, applying, and interpreting questionnaires. Additionally, using complementary assessment methods, such as clinical interviews, could be considered to provide a more comprehensive understanding of a patient’s condition.

Beyond these practical considerations, the substantial overlap between tinnitus distress and depressive symptom questionnaires may itself carry broader clinical meaning. Depressive symptom questionnaires are designed to capture depressive symptomatology specifically, and their overlap with tinnitus distress questionnaires suggests that tinnitus-related distress and depressive symptoms may be difficult to disentangle. This is further complicated by the fact that many tinnitus distress questionnaire items assess generic distress domains combined with a causal attribution to tinnitus, for example ‘because of your tinnitus.’ Respondents may not be able to fully disentangle the causes of their distress, which may stem from multiple sources, meaning tinnitus may become the label through which broader distress is interpreted and reported. High scores on both questionnaire types may therefore not only reflect co-occurring tinnitus-related distress and depressive symptoms, but may also indicate a transdiagnostic affective distress state, meaning an emotional distress process not specific to either symptom domain alone, in which scores on tinnitus distress questionnaires may partly reflect a broader shared underlying process. This has implications for how psychological tinnitus treatments are understood and evaluated, as such treatments may not only target tinnitus-related distress but also this broader affective distress state.

Several considerations should be taken into account when using the current content analysis to guide the selection of assessment tools. While the ICF is a standardized and internationally recognized framework ensuring a systematic examination of the content of the questionnaires, linking items to this framework remains susceptible to interpretation, as reflected in differences in coding decisions across studies [46–48]. For example, for items with concepts related to hearing and listening contexts, we applied the distinction described in Granberg et al. (2014) [42], whereas other studies have used different classifications [46]. Another example concerns ICF category *b126 temperament and personality functions*, which was not applied in the current study because it refers to personality traits rather than changes in psychological state [47]. In previous studies, however, this category has been applied to concepts such as confidence [46,48], while other classifications of such concepts, including emotional functions, have also been reported [47].

Moreover, ICF coding decisions may be influenced by the purpose of the analysis. Given the aim of the current study to compare tinnitus distress and depressive symptom questionnaires, items in which tinnitus was the direct object of the concept being measured were primarily linked to *b240 sensations associated with hearing and vestibular function*. As some of these items may alternatively be interpreted as measuring more general underlying processes, an alternative coding was applied in a sensitivity analysis. Under the alternative coding, overlap with the depressive symptom questionnaires increased for several tinnitus distress questionnaires. Particularly relevant in light of the findings of the main analysis were the increases in overlap for the TQ, from 11.54–26.92% to 21.15–42.31%, and for the TFI, from 16.00–36.00% to 20.00–48.00%. As a result, although the TQ and TFI remained among the tinnitus distress questionnaires showing lower degrees of overlap with the depressive symptom questionnaires, their overlap became more comparable to that of several other tinnitus distress questionnaires. Despite these changes, the overall pattern across questionnaires was largely maintained, with the TRQ continuing to show the highest degree of overlap and the overall conclusion of substantial content overlap remaining unchanged.

An additional consideration concerns the specificity and coverage of the ICF when applied to questionnaire content in the present study. For instance, the category *emotional functions* does not distinguish between different emotional states, and therefore items linked to this category encompass a wide range of emotional experiences. This broad categorization means that overlap is calculated based on this general concept, even when items may refer to different types of emotions. Furthermore, there are also concepts, such as suicidal thoughts or ideations, that are not currently covered in the ICF. These limitations underscore the framework’s inability to fully capture emotional and psychological symptoms represented in the questionnaires included in this study, emphasizing the potential value for an additional classification to capture these dimensions more accurately. A related issue was highlighted by the sensitivity analysis, in which items describing a person’s perceived capacity to cope with or control tinnitus could not be linked to an alternative ICF category and were therefore coded as *not covered*. The ICF framework describes what people experience in terms of functioning and disability, but does not distinguish between symptom descriptors and underlying processes such as cognitive appraisals or coping strategies, which limits the extent to which the current analysis can inform our understanding of the mechanisms driving overlap.

Furthermore, while using second-level codes generally provided the optimal balance between specificity and practicality, it can still result in simplification. This may lead to an apparent overlap between items that don’t share the exact same concept, such as items within the category *energy and drive functions*, which encompass a range of concepts including appetite, energy level and motivation. For other categories however, third-level codes may create artificial distinctions between conceptually similar items, potentially leading to an underestimation of overlap. Third-level ICF category data are nonetheless provided in the supporting material (S1 and S2 Files for the third-level codes for each questionnaire item and S5 File for the amount of item overlap between tinnitus distress and depressive symptom questionnaires based on the third-level categorization) for those who wish to examine this overlap.

Finally, it should be noted that the predominance of the *body functions* component does not imply that tinnitus or depression are primarily physiological in origin, as within the ICF framework *body functions* includes broad mental functions as well. For tinnitus in particular, this is an important nuance, given that the severity of tinnitus-related distress is known to be largely determined by cognitive and emotional processes rather than by the characteristics of the percept itself [4]. The ICF classification does not make this distinction, which should be kept in mind when interpreting the distribution of items across ICF components.

Future research on the relationship between tinnitus and depression should account for the content overlap in questionnaires when designing study methodologies and analyzing results, allowing for a more nuanced understanding of this relationship. Ultimately, recognizing and accounting for this overlap can lead to more insight of the complex interplay between tinnitus and depression, enhancing both clinical care and research outcomes.

## Conclusion

The ICF-based content analysis of tinnitus distress and depressive symptom questionnaires revealed overlap in items regarding *energy and drive functions, sleep functions, attention functions, emotional functions, thought functions, interpersonal interactions and relationships,* and *recreation and leisure*. The analysis showed that the TQ had the lowest degree of overlap with the depressive symptom questionnaires, while the TRQ exhibited the highest degree of overlap. Among the depressive symptom questionnaires, the SDS showed the least overlap with the tinnitus distress questionnaires, whereas the DASS-42 depression subscale demonstrated the most overlap. The substantial overlap between tinnitus distress and depressive symptom questionnaires emphasizes the importance of carefully selecting assessment tools and interpreting their results, based on specific clinical or research goals. This content analysis can guide in making decisions about this selection.

## Supporting information

S1 File Coding of tinnitus distress questionnaire items. (XLSX)

S2 File Coding of depressive symptom questionnaire items. (XLSX)

S3 File Cross-tables comparing each tinnitus distress questionnaire with each depressive symptom questionnaire. (XLSX)

S4 File Sensitivity analysis with alternative coding of b240 items. (PDF)

S5 File Amount of item overlap between tinnitus distress questionnaires and depressive symptom questionnaires based on third-level ICF categorization. (PDF)
