# Supplementary material for: An ICF-based content analysis of the overlap between questionnaires assessing tinnitus distress and depressive symptoms
Source: PLoS One. 2026 Sep 21;21(9):e0358644. doi: 10.1371/journal.pone.0358644 (PMC13592705; doi:10.1371/journal.pone.0358644)
Supplement: Supplementary file 1 [file pone.0358644.s004.pdf]

Supporting material file 4. Sensitivity analysis with alternative coding of b240 items.

Table 4.1. Alternative coding of b240 items

**Questionnaire item number**	**Question**	**Sensitivity analysis: second-level code**	**Sensitivity analysis: third-level code**
TFI1	Over the PAST WEEK… What percentage of your time awake were you consciously AWARE OF your tinnitus?	b240 Sensations associated with hearing and vestibular functions	b2400 Ringing in ears or tinnitus
TFI2	Over the PAST WEEK… How STRONG or LOUD was your tinnitus?	b240 Sensations associated with hearing and vestibular functions	b2400 Ringing in ears or tinnitus
TFI3	Over the PAST WEEK… What percentage of your time awake were you ANNOYED by your tinnitus?	b152 Emotional functions	b1529 Emotional functions, unspecified
TFI4	Over the PAST WEEK… Did you feel IN CONTROL in regard to your tinnitus?	NC	NC
TFI5	Over the PAST WEEK… How easy was it for you to COPE with your tinnitus?	NC	NC
TFI6	Over the PAST WEEK… How easy was it for you to IGNORE your tinnitus?	b140 Attention functions	b1401 Shifting attention
TFI9	Over the PAST WEEK… How much did your tinnitus interfere with… Your ability to FOCUS ATTENTION on other things besides your tinnitus?	b140 Attention functions	b1401 Shifting attention
THI6	Do you complain a great deal about your tinnitus?	NC	NC
THI8	Do you feel as though you cannot escape your tinnitus?	NC	NC
THI18	Do you find it difficult to focus your attention away from your tinnitus and on other things?	b140 Attention functions	b1401 Shifting attention
THI19	Do you feel that you have no control over your tinnitus?	NC	NC
THI23	Do you feel that you can no longer cope with your tinnitus?	NC	NC
THI24	Does your tinnitus get worse when you are under stress?	b240 Sensations associated with hearing and vestibular functions	b2400 Ringing in ears or tinnitus
THQ3	My tinnitus has gotten worse over the years.	b240 Sensations associated with hearing and vestibular functions	b2400 Ringing in ears or tinnitus
THQ8	I think I have a healthy outlook on tinnitus.	NC	NC
THQ23	I find it difficult to explain what tinnitus is to others.	NC	NC
TQ1	I can sometimes ignore the noises even when they are there	b140 Attention functions	b1401 Shifting attention

TQ3	It’s unfair that I have to suffer with my noises	b152 Emotional functions	b1529 Emotional functions, unspecified
TQ5/mTQ1	I am aware of the noises from the moment I get up to the moment I sleep	b240 Sensations associated with hearing and vestibular functions	b2400 Ringing in ears or tinnitus
TQ6	Your attitude to the noises makes no difference to how it affects you	NC	NC
TQ7	Most of the time the noises are fairly quiet	b240 Sensations associated with hearing and vestibular functions	b2400 Ringing in ears or tinnitus
TQ10	The way the noises sound is really unpleasant	b240 Sensations associated with hearing and vestibular functions	b2400 Ringing in ears or tinnitus
TQ11	I feel I can never get away from the noises	NC	NC
TQ13	I worry whether I will be able to put up with this problem forever	b152 Emotional functions	b1529 Emotional functions, unspecified
TQ15	The noises are loud most of the time	b240 Sensations associated with hearing and vestibular functions	b2400 Ringing in ears or tinnitus
TQ21	There is very little one can do to cope with the noises	NC	NC
TQ23	When I feel low or pessimistic the noise seems worse	b240 Sensations associated with hearing and vestibular functions	b2400 Ringing in ears or tinnitus
TQ27	It will be dreadful if these noises never go away	b152 Emotional functions	b1529 Emotional functions, unspecified
TQ29	The noises seem to go right through my head	b240 Sensations associated with hearing and vestibular functions	b2400 Ringing in ears or tinnitus
TQ30	Almost all my problems are caused by these noises	NC	NC
TQ32	It’s the way you think about the noise – NOT the noise itself which makes you upset	NC	NC
TQ35/mTQ7	My noises are often so bad that I cannot ignore them	b140 Attention functions	b1401 Shifting attention
TQ40	I am able to forget about the noises when I am doing something interesting	b140 Attention functions	b1401 Shifting attention
TQ43/mTQ10	I often think about whether the noises will ever go away	NC	NC
TQ44	I can imagine coping with the noises	NC	NC

TQ45	The noises never ‘let up’	b240 Sensations associated with hearing and vestibular functions	b2400 Ringing in ears or tinnitus
TQ46	A stronger person might be better at accepting this problem	b152 Emotional functions	b1529 Emotional functions, unspecified
TQ47/mTQ11	I am a victim of my noises	b152 Emotional functions	b1529 Emotional functions, unspecified
TQ49	The noises are one of those problems in life you have to live with	NC	NC

Table 4.2. Amount of item overlap between tinnitus distress and depressive symptom questionnaires based on second-level ICF categorization with alternative coding of b240 items

	BDI-II		HADS-D		SDS		PHQ-9		CES-D		SCL-90-R		DASS-42	
**THI**	THI	BDI-II	THI	HADS-D	THI	SDS	THI	PHQ-9	THI	CES-D	THI	SCL-90	THI	DASS-42
	13/25	17/21	11/25	6/7	12/25	12/20	13/25	7/9	13/25	17/20	11/25	13/16	10/25	14/14
	52.00%	80.95%	44.00%	85.71%	48.00%	60.00%	52.00%	77.78%	52.00%	85.00%	44.00%	81.25%	40.00%	100.00%
**TQ**	TQ	BDI-II	TQ	HADS-D	TQ	SDS	TQ	PHQ-9	TQ	CES-D	TQ	SCL-90	TQ	DASS-42
	22/52	14/21	15/52	5/7	17/52	8/20	22/52	5/9	22/52	14/20	12/52	9/16	11/52	12/14
	42.31%	66.67%	28.85%	71.43%	32.69%	40.00%	42.31%	55.56%	42.31%	70.00%	23.08%	56.25%	21.15%	85.71%
**mTQ**	mTQ	BDI-II	mTQ	HADS-D	mTQ	SDS	mTQ	PHQ-9	mTQ	CES-D	mTQ	SCL-90	mTQ	DASS-42
	7/12	14/21	5/12	5/7	6/12	9/20	7/12	5/9	6/12	14/20	5/12	11/16	4/12	12/14
	58.33%	66.67%	41.67%	71.43%	50.00%	45.00%	58.33%	55.56%	50.00%	70.00%	41.67%	68.75%	33.33%	85.71%
**THQ**	THQ	BDI-II	THQ	HADS-D	THQ	SDS	THQ	PHQ-9	THQ	CES-D	THQ	SCL-90	THQ	DASS-42
	10/27	17/21	9/27	6/7	9/27	11/20	10/27	7/9	11/27	19/20	8/27	11/16	8/27	14/14
	37.04%	80.95%	33.33%	85.71%	33.33%	55.00%	37.04%	77.78%	40.74%	95.00%	29.63%	68.75%	29.63%	100.00%
**TRQ**	TRQ	BDI-II	TRQ	HADS-D	TRQ	SDS	TRQ	PHQ-9	TRQ	CES-D	TRQ	SCL-90	TRQ	DASS-42
	18/26	14/21	19/26	5/7	18/26	9/20	18/26	5/9	18/26	14/20	17/26	11/16	16/26	12/14
	69.23%	66.67%	73.08%	71.43%	69.23%	45.00%	69.23%	55.56%	69.23%	70.00%	65.38%	68.75%	61.54%	85.71%
**TFI**	TFI	BDI-II	TFI	HADS-D	TFI	SDS	TFI	PHQ-9	TFI	CES-D	TFI	SCL-90	TFI	DASS-42
	11/25	14/21	8/25	5/7	9/25	9/20	11/25	5/9	12/25	16/20	6/25	11/16	5/25	12/14
	44.00%	66.67%	32.00%	71.43%	36.00%	45.00%	44.00%	55.56%	48.00%	80.00%	24.00%	68.75%	20.00%	85.71%

BDI-II, Beck Depression Inventory-II; *HADS-D*, Hospital Anxiety and Depression Scale – depression subscale; *SDS*, Zung Self-Rating Depression Scale; *PHQ-9*, Patient Health Questionnaire-9; *CES-D*; Center for Epidemiologic Studies Depression Scale; *SCL-90-R*, Symptom Checklist-90-Revised; *DASS-42*, Depression Anxiety Stress Scale 42

Note: This table presents the item overlap between various tinnitus distress and depressive symptom questionnaires based on second-level ICF categorization with alternative coding of b240 items. For example, 13 out of 25 items (52%) from the THI overlap with the BDI, and 17 out of 21 items (80.95%) from the BDI-II overlap with the THI.
