# Supplementary material for: An ICF-based content analysis of the overlap between questionnaires assessing tinnitus distress and depressive symptoms
Source: PLoS One. 2026 Sep 21;21(9):e0358644. doi: 10.1371/journal.pone.0358644 (PMC13592705; doi:10.1371/journal.pone.0358644)
Supplement: Supplementary file 2 [file pone.0358644.s005.pdf]

Supporting material file 5. Amount of item overlap between tinnitus distress and depressive symptom questionnaires based on third-level ICF categorization

	**BDI-II**		**HADS-D**		**SDS**		**PHQ-9**		**CES-D**		**SCL-90-R**		**DASS-42**	
	THI	BDI-II	THI	HADS-D	THI	SDS	THI	PHQ-9	THI	CES-D	THI	SCL-90	THI	DASS-42
**THI**	11/25	14/21	10/25	5/7	10/25	7/20	12/25	6/9	9/25	11/20	10/25	10/16	9/25	11/14
	44.00%	66.67%	40.00%	71.43%	40.00%	35.00%	48.00%	66.67%	36.00%	55.00%	40.00%	62.50%	36.00%	78.57%
	TQ	BDI-II	TQ	HADS-D	TQ	SDS	TQ	PHQ-9	TQ	CES-D	TQ	SCL-90	TQ	DASS-42
**TQ**	8/52	11/21	9/52	4/7	9/52	7/20	11/52	5/9	8/52	12/20	7/52	9/16	6/52	11/14
	15.38%	52.38%	17.31%	57.14%	17.31%	35.00%	21.15%	55.56%	15.38%	60.00%	13.46%	56.25%	11.54%	78.57%
	mTQ	BDI-II	mTQ	HADS-D	mTQ	SDS	mTQ	PHQ-9	mTQ	CES-D	mTQ	SCL-90	mTQ	DASS-42
**mTQ**	4/12	12/21	3/12	4/7	3/12	6/20	5/12	5/9	3/12	11/20	3/12	9/16	3/12	11/14
	33.33%	57.14%	25.00%	57.14%	25.00%	30.00%	41.67%	55.56%	25.00%	55.00%	25.00%	56.25%	25.00%	78.57%
	THQ	BDI-II	THQ	HADS-D	THQ	SDS	THQ	PHQ-9	THQ	CES-D	THQ	SCL-90	THQ	DASS-42
**THQ**	9/27	14/21	7/27	4/7	8/27	7/20	10/27	7/9	8/27	13/20	8/27	10/16	7/27	11/14
	33.33%	66.67%	25.93%	57.14%	29.63%	35.00%	37.04%	77.78%	29.63%	65.00%	29.63%	62.50%	25.93%	78.57%
	TRQ	BDI-II	TRQ	HADS-D	TRQ	SDS	TRQ	PHQ-9	TRQ	CES-D	TRQ	SCL-90	TRQ	DASS-42
**TRQ**	17/26	13/21	15/26	4/7	17/26	8/20	16/26	4/9	16/26	12/20	15/26	9/16	15/26	11/14
	65.38%	61.90%	57.69%	57.14%	65.38%	40.00%	61.54%	44.44%	61.54%	60.00%	57.69%	56.25%	57.69%	78.57%
	TFI	BDI-II	TFI	HADS-D	TFI	SDS	TFI	PHQ-9	TFI	CES-D	TFI	SCL-90	TFI	DASS-42
**TFI**	6/25	13/21	4/25	4/7	5/25	7/20	7/25	5/9	6/25	14/20	4/25	10/16	4/25	11/14
	24.00%	61.90%	16.00%	57.14%	20.00%	35.00%	28.00%	55.56%	24.00%	70.00%	16.00%	62.50%	16.00%	78.57%

BDI-II, Beck Depression Inventory-II; *HADS-D*, Hospital Anxiety and Depression Scale – depression subscale; *SDS*, Zung Self-Rating Depression Scale; *PHQ-9*, Patient Health Questionnaire-9; *CES-D*; Center for Epidemiologic Studies Depression Scale; *SCL-90-R*, Symptom Checklist-90-Revised; *DASS-42*, Depression Anxiety Stress Scale 42

Note: This table presents the item overlap between various tinnitus distress and depressive symptom questionnaires based on third-level ICF categorization. For example, 11 out of 25 items (44%) from the THI overlap with the BDI, and 14 out of 21 items (66.67%) from the BDI-II overlap with the THI.
