# Supplementary material for: An ICF-based content analysis of the overlap between questionnaires assessing tinnitus distress and depressive symptoms
Source: PLoS One. 2026 Sep 21;21(9):e0358644. doi: 10.1371/journal.pone.0358644 (PMC13592705; doi:10.1371/journal.pone.0358644)
Supplement: Supplementary file 3 [file pone.0358644.s007.docx]

Dear dr. Bojana Bukurov, reviewer #1 and reviewer #2,

We thank you for your careful consideration of our manuscript and for providing constructive feedback. We have revised our manuscript to address the points raised.

Comments from the academic editor
Regarding the comments from the Academic Editor, we have revised the manuscript to comply with PLOS ONE’s style requirements (comment 1). In addition, we have added captions for the supporting information files at the end of the manuscript and updated the corresponding in-text citations (comment 2). Comment 3 is not applicable, as the reviewers did not recommend citing any specific additional published work.

Comments from reviewer #1:
We would like to thank reviewer #1 for their detailed and insightful feedback on our manuscript. For readability, we have summarized the comments below, followed by our point-by-point responses.

1. **Clarify construct validity: “depression” and “tinnitus” are heterogeneous descriptive labels, not unitary conditions**
   Comment: The reviewer emphasized that both depression and tinnitus are heterogeneous descriptive labels rather than unitary conditions. Regarding tinnitus specifically, the reviewer stressed the importance of distinguishing between the tinnitus percept, tinnitus-related distress as operationalized by questionnaires, and hearing loss as an audiological correlate. The reviewer recommended that findings be consistently interpreted as overlap between questionnaire operationalizations rather than between underlying conditions.
   Response: We agree with this distinction and have added clarification to the introduction distinguishing between tinnitus as a self-reported percept and tinnitus-related distress as assessed by questionnaires (lines 103-107 in the clean version of the manuscript). We further specified that overlap should be interpreted at the level of questionnaire operationalization rather than as evidence of overlap between underlying conditions (lines 108-110, 353).
   Regarding the point on hearing loss as an audiological correlate that is sometimes used as a proxy for tinnitus: we agree this is an important conceptual issue in the broader literature. However, as our study is confined to a content analysis of self-report questionnaires and does not involve audiological measures, this concern does not directly apply to our methodology and was therefore not further addressed in the manuscript.
2. **Foreground the attribution problem: “because of tinnitus” does not measure cause and may inflate overlap by design**
   **Comment:** The reviewer noted that many tinnitus questionnaires assess generic distress domains and then add causal attribution via wording like 'because of tinnitus,' which does not measure distress caused by tinnitus in a causal sense but rather the respondent's attribution of distress to the tinnitus percept. The reviewer argued this can produce conceptual circularity and mechanically inflate overlap with depression scales, and recommended treating attribution-laden item wording as a named interpretive limitation.
   Response: We have now added some information in the discussion acknowledging attribution-laden item wording in tinnitus questionnaires and its implications (lines 446-451).
3. **“Body functions” dominance (Table 2) requires a more cautious interpretation**
   Comment: The reviewer raised concerns about the predominance of body functions in Table 2, noting that ICF places mental functions within body functions and that this should not imply physiological etiology. More specifically, the reviewer was concerned that appraisal, coping, and attention-regulation items were coded under sensory categories such as b2400, making tinnitus questionnaires appear more sensory than their content warrants. The reviewer requested explicit decision rules for these ambiguous items and a sensitivity analysis recoding them under cognitive/emotional categories.
   Response: We have added a clarification in the discussion about the domain body functions (lines 358-360 & lines 512-519). Regarding the coding of items under b2400 ringing in ears or tinnitus, this was applied only to items whose core concept is inextricably linked to the tinnitus experience and cannot be separated from it, as is now also mentioned in the methods section (lines 190-201). We therefore did not perform the suggested sensitivity check, as recoding these items under mental function categories would suggest overlap with depression questionnaires where there is none, given that their content is inextricably linked to the tinnitus experience itself.
4. **Methodological transparency: rater background and reliability are essential**
   **Comment:** The reviewer highlighted the importance of reporting rater backgrounds, coding procedures, and reliability measures, noting that without reliability evidence, overlap results risk being interpreted as reflecting what these particular coders decided rather than a stable property of questionnaire content.
   Response: We agree with the reviewer that initiatives to make the work reproducible should be encouraged and thank them for all mentioned suggestions. Several revisions have been made to address these concerns regarding methodological transparency. We have added the rater backgrounds (lines 182-183 & 214-215) and reproducible decision rules (lines 188-212 & lines 226-232) to the method section. Furthermore, to enhance transparency we reported how many items were discussed with the third reviewer to reach consensus, including further specification of the nature of these items (lines 214-225).
5. **Coarse second-level categories may overstate overlap**
   Comment: The reviewer raised concerns that broad second-level ICF categories such as b152 emotional functions may generate overlap even when items reflect different phenomena, such that high overlap may reflect category breadth rather than meaningful construct similarity. The reviewer suggested a sensitivity analysis using third-level categories and reporting overlap under both the current criterion and a stricter criterion incorporating item intent.
   Response: We acknowledge that broad second-level ICF categories such as b152 emotional functions may generate overlap based on category breadth rather than meaningful construct similarity, which is already discussed in the limitations section (lines 501-507). At the same time, for other categories, third-level codes may create artificial distinctions between conceptually similar items, potentially leading to an underestimation of overlap. However, for readers who wish to examine the overlap at a more granular level, we have included the third-level ICF category overlap as supplementary material, which is briefly referred to in the limitations section (lines 507-511).
6. **Distinguish “WHAT symptoms” from “WHY/mediating processes”**
   **Comment:** The reviewer recommended explicitly distinguishing between 'what' symptom descriptors such as low mood or sleep disturbance, and 'why' mediating processes such as cognitive appraisals, perceived control, coping strategies, and attentional biases. The reviewer argued that overlap at the 'what' level may reflect shared nonspecific distress domains, whereas overlap at the 'why' level would suggest shared interpretive and appraisal processes relevant to tinnitus distress maintenance.
   Response: We agree this distinction would add valuable insight, but the ICF framework is designed to describe what people experience in terms of functioning and disability, and does not capture why or through what processes symptoms arise and are maintained. We have acknowledged this as a limitation in the discussion (lines 496-500), and consider it an interesting direction for future research.
7. **Interpret the DASS finding beyond “avoid these scales”: clinical meaning of “overlap” may be relevant for emotion/distress processes, not “tinnitus”**
   **Comment:** Beyond the psychometric implications, the reviewer argued that high overlap, particularly with the DASS, may reflect that both sets of questionnaires are sampling a transdiagnostic affective distress dimension rather than two separable conditions. The reviewer encouraged discussing the clinical implications of this, suggesting that tinnitus-related suffering may be embedded in broader emotion regulation and stress vulnerability processes rather than being a tinnitus-specific symptom domain.
   Response: We have expanded the discussion to include a clinical process interpretation of the overlap beyond the psychometric implications (lines 442-457).
8. **Minor comments / clarifications**
   Comment: The reviewer requested additional justification for omitting the perspective and response option linking steps, asked for a legend to be added to Supplementary Material 2, and suggested consistently using terms such as 'tinnitus distress questionnaires' and 'depressive symptom questionnaires' to avoid reification.
   Response: Regarding the minor clarifications; we have expanded on why we did not apply the perspective and response option linking rules in the methods (lines 172-177). We also added a legend to supplementary material 2 and used the terms tinnitus distress questionnaires and depressive symptom questionnaires when appropriate.

Reviewer #2:
We thank Reviewer #2 for their review of our manuscript and for highlighting its clinical relevance and practical applicability.

We appreciate the opportunity to revise our manuscript and hope that the changes and clarifications address the concerns raised. Please let us know if any further modifications are required.

Sincerely,

On behalf of all authors,

Denise Fuchten
