# Supplementary material for: An ICF-based content analysis of the overlap between questionnaires assessing tinnitus distress and depressive symptoms
Source: PLoS One. 2026 Sep 21;21(9):e0358644. doi: 10.1371/journal.pone.0358644 (PMC13592705; doi:10.1371/journal.pone.0358644)
Supplement: Supplementary file 4 [file pone.0358644.s008.docx]

Dear reviewer #1,

We thank you for this careful and constructive re-review, and for confirming that several revisions move the manuscript in the intended direction. We have revised our manuscript to address the points raised, and address each of the points below following your original numbering. For brevity, quotes from the comments are excerpted to the part specifying the concern or requested action.

- **Comments 1, 2, 6 and 7**

Reviewer: *“Points 1, 2, 6, and 7 are satisfactorily addressed and meaningfully strengthen the manuscript's conceptual framing in the direction I was advocating […].”*

Response: We are happy to hear that these points are adequately addressed.

- **Comment 3**

Reviewer: *"[…] Whether an item like 'ability to ignore tinnitus' is best understood as a description of the sensory percept or as a description of an attentional/coping process is exactly the question in dispute. […] I had asked for a sensitivity analysis recoding these ambiguous items under cognitive/emotional/attentional categories; the authors declined to run it, offering only a supplementary third-level breakdown that does not address this specific ambiguity. […] I'd ask the authors to either run the requested sensitivity analysis or explicitly flag, as a stated limitation with the TQ result named, that the low overlap estimate is partly contingent on this coding decision."*

Response: Thank you for this detailed feedback. We have run the requested sensitivity analysis, and reported on this in the methods (lines 259-272 and 286-291 in the manuscript with tracked changes), results (lines 412-423) and discussion (571-585).

- **Comment 4**

Reviewer: *“[…] At minimum, I'd ask for reporting of raw agreement before adjudication (percentage agreement, even without chance-correction, is better than nothing), since it directly bears on how much weight the overlap percentages in Table 4 can bear.”*
Response: Thank you for this suggestion. We have added the requested raw agreement statistic to the result section (lines 305-313), and adjusted the method section accordingly (lines 292-299).

- **Comment 5**

Reviewer: “*Acknowledged as a limitation, and third-level data are now provided as Supplementary Material 3 for interested readers. Given the practical constraints, reporting overlap only at the second level in the main text while providing third-level data as supplementary material is an acceptable compromise, though it does mean the headline percentages in the abstract still carry the coarse-category inflation risk without a bounded estimate alongside them*.”
Response: We thank you for confirming that this is an acceptable compromise given the practical constraints. We have now added "at the second-level ICF category" next to the reported percentages in the abstract's results sentence to make the source clear to the reader.

- **Comment 8**

Reviewer: “*The tracked-changes version still contains leftover artifacts from incomplete find-and-replace (e.g., "depression depressive symptom questionnaires," "the depressionse questionnaires," a stray "6070%" in the CES-D range) that need to be cleaned up in the final clean copy before typesetting; these look like copy-editing residue rather than substantive issues.”*
Response: We thank the reviewer for flagging these. These were residual artifacts from the tracked-changes file and have been corrected in the clean copy submitted with this revision.

We appreciate the opportunity to revise our manuscript and hope that the changes and clarifications address the concerns raised. Please let us know if any further modifications are required.

Sincerely,

On behalf of all authors,

Denise Fuchten
