# Supplementary material for: An ICF-based content analysis of the overlap between questionnaires assessing tinnitus distress and depressive symptoms
Source: PLoS One. 2026 Sep 21;21(9):e0358644. doi: 10.1371/journal.pone.0358644 (PMC13592705; doi:10.1371/journal.pone.0358644)
Supplement: Peer review history

# Peer review: Decision Letter 0

**DOI:** 10.1371/journal.pone.0358644.r001

**Authors:** Bojana Bukurov

22 Feb 2026

Dear Dr. Fuchten,

Thank you for submitting your manuscript to PLOS ONE. After careful consideration, we feel that it has merit but does not fully meet PLOS ONE’s publication criteria as it currently stands. Therefore, we invite you to submit a revised version of the manuscript that addresses the points raised during the review process.

Please submit your revised manuscript by Apr 08 2026 11:59PM. If you will need more time than this to complete your revisions, please reply to this message or contact the journal office at plosone@plos.org. When you're ready to submit your revision, log on to https://www.editorialmanager.com/pone/ and select the 'Submissions Needing Revision' folder to locate your manuscript file.

- A letter that responds to each point raised by the academic editor and reviewer(s). You should upload this letter as a separate file labeled 'Response to Reviewers'.
- A marked-up copy of your manuscript that highlights changes made to the original version. You should upload this as a separate file labeled 'Revised Manuscript with Track Changes'.
- An unmarked version of your revised paper without tracked changes. You should upload this as a separate file labeled 'Manuscript'.

If you would like to make changes to your financial disclosure, please include your updated statement in your cover letter. Guidelines for resubmitting your figure files are available below the reviewer comments at the end of this letter.

If applicable, we recommend that you deposit your laboratory protocols in protocols.io to enhance the reproducibility of your results. Protocols.io assigns your protocol its own identifier (DOI) so that it can be cited independently in the future. For instructions see: https://journals.plos.org/plosone/s/submission-guidelines#loc-laboratory-protocols. Additionally, PLOS ONE offers an option for publishing peer-reviewed Lab Protocol articles, which describe protocols hosted on protocols.io. Read more information on sharing protocols at https://plos.org/protocols?utm_medium=editorial-email&utm_source=authorletters&utm_campaign=protocols.

We look forward to receiving your revised manuscript.

Kind regards,

Bojana Bukurov, M.D., Ph.D.

Academic Editor

PLOS One

Journal Requirements:

When submitting your revision, we need you to address these additional requirements.

1. Please ensure that your manuscript meets PLOS ONE's style requirements, including those for file naming. The PLOS ONE style templates can be found at

https://journals.plos.org/plosone/s/file?id=wjVg/PLOSOne_formatting_sample_main_body.pdf and

https://journals.plos.org/plosone/s/file?id=ba62/PLOSOne_formatting_sample_title_authors_affiliations.pdf

2. Please include captions for your Supporting Information files at the end of your manuscript, and update any in-text citations to match accordingly. Please see our Supporting Information guidelines for more information: http://journals.plos.org/plosone/s/supporting-information.

3. If the reviewer comments include a recommendation to cite specific previously published works, please review and evaluate these publications to determine whether they are relevant and should be cited. There is no requirement to cite these works unless the editor has indicated otherwise.

[Note: HTML markup is below. Please do not edit.]

Reviewers' comments:

Reviewer's Responses to Questions

**Comments to the Author**

1. Is the manuscript technically sound, and do the data support the conclusions?

Reviewer #1: Yes

Reviewer #2: Yes

**********

2. Has the statistical analysis been performed appropriately and rigorously?

Reviewer #1: Yes

Reviewer #2: Yes

**********

3. Have the authors made all data underlying the findings in their manuscript fully available?

The PLOS Data policy

Reviewer #1: Yes

Reviewer #2: Yes

**********

4. Is the manuscript presented in an intelligible fashion and written in standard English?

Reviewer #1: Yes

Reviewer #2: Yes

**********

Reviewer #1: Overall assessment

This manuscript tackles an important and under-addressed problem: content overlap between tinnitus questionnaires and depression questionnaires, which can inflate correlations and complicate interpretation. The overall approach—using ICF linking to make overlap visible and quantifiable—is valuable, and I appreciate that the paper explicitly highlights heterogeneity in both “tinnitus questionnaires” and “depression questionnaires.” The attempt to map content across commonly used instruments is useful for both research and clinical purposes, particularly in a field that is often conceptually inconsistent in how “tinnitus,” “distress,” “audiological characteristics,” and “hearing loss” are used and sometimes conflated.

That said, I have major concerns that the manuscript—despite aiming to reduce conceptual confusion—still risks perpetuating it, chiefly by (i) reifying “tinnitus” and “depression” as quasi-medical “conditions,” and (ii) drawing strong practical conclusions from an overlap metric that is highly sensitive to coder interpretation and to the coarseness of second-level ICF categories. In particular, tinnitus instruments often measure generic distress domains plus a self-attribution (“because of tinnitus”), which may be a key mechanism driving apparent “overlap” and inflated associations. This matters because it affects how readers interpret your findings and the implications for clinical trials.

Below I outline concrete revisions that would substantially strengthen the work.

1. Clarify construct validity: “depression” and “tinnitus” are heterogeneous descriptive labels, not unitary conditions

The manuscript sometimes implies that tinnitus and depression are two separable “conditions” whose “shared symptomatology” can be quantified. While the overestimation argument is valid and welcome, the paper would benefit from an explicit clarification that:

“Depression” is heterogeneous even descriptively. Depression questionnaires differ substantially in how they weight and combine somatic/vegetative symptoms (sleep, energy, appetite), cognitive themes (worthlessness, hopelessness), affective/emotional states (sadness, anhedonia), and behavioral patterns (withdrawal). Even at a checklist level, “depression” reflects a family of partially overlapping symptom constellations (sometimes partly opposing: agitation vs slowing; insomnia vs hypersomnia; appetite loss vs overeating; emotional numbness vs tearfulness). Therefore, “overlap” will depend heavily on which symptom dimensions a given depression instrument samples.

“Tinnitus” is not a single disease entity with a defined pathophysiology. The manuscript would benefit from a more explicit conceptual clarification of what is meant by “tinnitus” throughout. In current clinical and research practice, “tinnitus” is frequently used to refer interchangeably to: (i) the self-reported percept (auditory sensation), (ii) tinnitus-related distress/impact as measured by questionnaires, and (iii) hearing loss/audiological findings used as correlates or proxies. However, these are not interchangeable entities.

Importantly, for most patients there is no objective biomarker or definitive pathophysiological signature that allows clinicians to “find” tinnitus in the way that would be implied by a disease-entity framing. In practice, tinnitus remains primarily accessible via self-report, whereas hearing loss is an audiological characteristic that may co-occur and sometimes be used—implicitly or explicitly—as a proxy for tinnitus. This proxying can inadvertently perpetuate conceptual confusion and complicate interpretation of questionnaire overlap, because distress scales can become treated as measures of “tinnitus” rather than measures of distress and attribution in the context of a self-reported percept. Without explicitly separating (i) tinnitus percept/self-report, (ii) tinnitus-related distress/impact scores, and (iii) hearing loss as a co-occurring factor (sometimes used as a proxy), the paper risks conflating these layers and encouraging readers to interpret overlap as overlap between “conditions,” rather than overlap between measurement instruments and attributional item content.

Suggested revisions:

Add a short paragraph in the Introduction clarifying these distinctions (tinnitus percept vs distress/impact vs hearing loss), and ensure the Discussion consistently interprets findings as overlap between questionnaire operationalizations, not between underlying disease entities. Moreover, if applicable, explicitly state that hearing loss should not be treated as a proxy marker for tinnitus in the interpretation of overlap. Throughout the manuscript, please distinguish clearly between tinnitus as a self-reported percept and tinnitus-related distress as operationalized by questionnaires or "hearing loss" as an audiological correlate that should not be (but is often treated) treated as a proxy for tinnitus itself.

2. Foreground the attribution problem: “because of tinnitus” does not measure cause and may inflate overlap by design

A key point that is currently not made explicitly enough is that many tinnitus questionnaires assess generic distress domains (sleep, concentration, irritability, sadness, enjoyment of life) and then add causal attribution (e.g., “because of tinnitus”). This does not measure distress caused by tinnitus in a validated causal sense; rather, it measures the respondent’s attribution of distress of potentially multiple origins to the tinnitus percept.

Respondents are not necessarily able to validly disentangle causes of sadness, irritability, or sleep problems (work stress, relationships, health, personality vulnerability, hearing difficulties, etc.) and then allocate a unique portion to tinnitus. The item becomes a measure of attribution of distress to a salient symptom, which can produce conceptual circularity: tinnitus becomes the label through which generic distress is interpreted and reported—mechanically increasing overlap with depression scales and inflating associations.

Suggested revisions:

Treat “attribution-laden item wording” as a named interpretive limitation and discuss how it may (i) contribute to overlap and (ii) constrain conclusions about distinguishing tinnitus and depression in trials.

3. “Body functions” dominance (Table 2) requires a more cautious interpretation

My concern here is not about the ICF hierarchy per se (ICF places mental functions within “body functions”), but about the interpretation the text invites—namely, that tinnitus and depression are primarily bodily/physiological problems. This is precisely where the tinnitus field is conceptually vulnerable (tinnitus percept vs distress response; bottom-up contributors vs top-down appraisal/meaning).

This issue becomes concrete in the item-level linking tables: several appraisal/regulation/strategy items (e.g., perceived control, coping, ignoring tinnitus, shifting attention away) are coded as “Body functions,” and in some cases under sensory categories such as b2400 “Ringing in ears or tinnitus.” These are not straightforward sensory descriptors; they primarily reflect top-down attention allocation, perceived control, coping capacity, and self-regulation. Coding them as sensory tinnitus content risks making tinnitus questionnaires appear more “bodily/sensory” than their face-valid content and may contribute to the “body functions dominance” conclusion as a coding artifact.

Suggested revisions:

Reword the Table 2/Discussion interpretation to explicitly state that “body functions” includes broad mental functions and does not imply physiological etiology.

Provide explicit decision rules for coding control/coping/attention-deployment items (sensory percept vs mental functions vs activities/participation).

Add a small sensitivity check: recode a subset of clear emotion regulation/appraisal items (control/cope/ignore/attention-away) under a cognition/emotion/behavioural strategy framing (even if still within ICF mental functions) and show whether Table 2 distributions and overlap percentages change.

4) Methodological transparency: rater background and reliability are essential

The manuscript states that inter-rater reliability was not calculated due to recoding and early ambiguities. This is a serious limitation for a study where overlap estimates depend directly on item-to-category assignments.

Suggested revisions:

Report rater background/roles (audiology, psychology, mixed) because professional perspective plausibly shapes linking decisions.

Report initial agreement (prior to consensus/recoding), even as simple percent agreement by chapter or by second-level category.

Provide reproducible decision rules for resolving ambiguous cases (e.g., how appraisal items were placed in b240 vs b152 vs b160).

Consider a small robustness check: a third rater codes a stratified subset of ambiguous items (e.g., 10–20%) and report agreement.

Without some reliability evidence, the overlap results risk being interpreted as “what these particular coders decided” rather than a stable property of questionnaire content.

5) Coarse second-level categories may overstate overlap (and need sensitivity analysis)

Because overlap is evaluated at second-level ICF categories, broad bins (e.g. b152 Emotional functions) can generate overlap even when items reflect different emotional phenomena or item intent. This matters especially when a scale is concentrated in very few bins (e.g., a depression subscale mapping largely to b152/b130). In that case, high overlap may reflect category breadth rather than meaningful construct similarity.

Suggested revisions:

Add a sensitivity analysis using third-level categories where feasible (e.g., b2400 vs b240), or supplement with a parallel “subtheme” classification to show how much overlap is driven by category granularity.

Report overlap in two ways:

- current criterion (“any shared second-level category”), and

- an exploratory stricter criterion incorporating item intent (e.g., affective state vs cognitive appraisal vs behavioral withdrawal vs functional interference).

6) Distinguish “WHAT symptoms” from “WHY/mediating processes”

The paper would benefit from explicitly distinguishing:

“WHAT” symptom descriptors (e.g., low mood, sleep disturbance, low energy), versus

“WHY”/mediating processes (cognitive appraisals, perceived control, coping strategies, attentional biases, avoidance/withdrawal behaviors).

Overlap at the “WHAT” level may reflect shared nonspecific distress domains; overlap at the “WHY” level would suggest shared interpretive/appraisal processes relevant to tinnitus distress (or “depression”) maintenance. This would align strongly with your stated goals and with contemporary clinical models of symptom chronification.

Suggested revisions:

Add a secondary (even qualitative) layer of classification separating symptom descriptors vs appraisal/interpretation vs behavior/coping/avoidance.

7) Interpret the DASS finding beyond “avoid these scales”: clinical meaning of “overlap” may be relevant for emotion/distress processes, not “tinnitus”

The high overlap between the DASS-42 depression subscale and several tinnitus questionnaires is an important psychometric warning for trial design (measurement non-independence as you pose it now). However, I encourage the authors to also interpret this finding more explicitly in clinical-process terms, rather than only as a methodological recommendation to avoid certain instrument combinations:

Many tinnitus questionnaires include items that are either (a) generic distress content that is attributed to tinnitus (“because of tinnitus”), or (b) appraisal/regulation content (e.g., coping, control, annoyance, attention capture). The DASS depression subscale places comparatively strong emphasis on negative emotional states / distress language (and less on some of the more “structural” cognitive or behavioral components emphasized by other depression scales). When such an emotional-state–weighted scale overlaps strongly with tinnitus distress instruments, this may indicate something clinically relevant:

The shared variance is not necessarily “tinnitus and depression as two diseases overlap,” but rather that both sets of questionnaires are sampling a transdiagnostic affective distress dimension (e.g., negative affect, emotional distress, stress-related arousal, reduced positive affect) that is clinically central to tinnitus distress persistence and chronification.

In other words, the DASS finding can be framed as a pointer toward what maintains suffering after tinnitus onset: the problem may not be the sensory percept per se, but the emotional and interpretive response to the percept (threat appraisal, catastrophic meaning, helplessness/lack of control, attentional capture, avoidance/safety behaviors), which then generalizes into broader distress and functional impact and appears best “grasped” by DASS content (or at least lend itself to the highest confounding with some tinnitus-related distress questionnaires). High overlap would therefore be consistent with a clinical formulation where tinnitus-related suffering is embedded in broader emotion regulation and stress vulnerability processes, rather than being a tinnitus-specific “symptom domain.” It may be important to state explicitly that psychologically focused interventions do not necessarily “improve tinnitus” (as a percept) and may not map cleanly onto “tinnitus-related distress” scores when those scores rely on simplified causal attributions (“because of tinnitus”). Clinically, many such interventions target broader, transdiagnostic processes—emotion regulation and acceptance, appraisal and threat interpretation, attentional deployment, coping/avoidance patterns, and sleep/arousal regulation—that shape how sensory experiences (including but not limited to tinnitus) are perceived, interpreted, and managed. From this perspective, the outcome is less a disease-like “condition treated → symptom removed” trajectory and more a development of adaptive stimulus processing and coping in person-in-context, where distress and functional impact can improve even if tinnitus loudness or awareness does not.

Suggested revisions for the Discussion:

Please clearly separate two interpretations:

- Psychometric interpretation (what your data strictly show): The overlap implies measurement non-independence. Using TRQ alongside DASS-42 (or other highly overlapping pairs) can inflate correlations and obscure whether a trial has changed tinnitus-related impact versus general distress content.

- Clinical-process interpretation (hypothesis generated by your findings): High overlap, especially with an emotion-state–weighted instrument like DASS, may indicate that tinnitus distress questionnaires partly capture transdiagnostic emotional distress and attribution/appraisal processes. Clinically, this could support a broader focus on emotion regulation, threat appraisal, helplessness/control beliefs, attentional deployment, and avoidance behaviors, rather than a mechanistic emphasis on “tinnitus” as a sensory entity. This is not proven by your content analysis, but it is a plausible and clinically useful implication that aligns with the aim of clarifying what questionnaire scores can and cannot mean. If a patient (or trial cohort) scores high on both a tinnitus distress measure and DASS depression, it may be clinically misleading to interpret this as “tinnitus caused depression” or as two separable conditions. It may instead reflect a shared distress/emotion process where the tinnitus percept becomes incorporated into broader emotional vulnerability and stress appraisal. This would argue for interventions that directly target those processes (e.g., transdiagnostic CBT/ACT elements, emotion-focused work, attention training, sleep and arousal regulation), rather than assuming that reducing the percept alone is the key route to symptom relief.

Minor comments / clarifications

You did not apply two steps from the 2019 linking rules (perspective; response options). Please justify more explicitly why these steps are “not relevant,” or acknowledge that omitting them may limit interpretation.

Add a short legend to Supplementary Material 2 explaining how to read the cross-tables (what rows/columns represent; what a filled cell means).

Consider consistently using the terms “tinnitus distress questionnaires” and “depressive symptom questionnaires” to avoid reification.

Recommendation

Major revision. The manuscript has real potential and addresses an important interpretive problem. To fully deliver on the promise of reducing conceptual confusion, it needs:

Explicit construct clarification of “tinnitus” and “depression” as heterogeneous descriptive labels, and a clearer statement of what the questionnaires can (and cannot) represent (percept vs distress/impact vs hearing loss proxies; symptom descriptors vs mediating processes).

A stronger, more explicit discussion of attribution-laden item wording in many tinnitus instruments (“because of tinnitus”) as a likely driver of apparent overlap and inflated associations—i.e., that such items may index attribution/interpretation of distress rather than a tinnitus-specific causal construct.

Greater methodological transparency and robustness around the coding process (rater background/roles, decision rules for ambiguous items, and at least partial reliability reporting or sensitivity analyses), particularly for items currently classified under “body functions” or even sensory tinnitus categories despite clearly tapping appraisal/coping/attention regulation.

Additional brief clinical considerations: While this work is methodological, its implications are clinically consequential. If “tinnitus,” “tinnitus distress,” hearing-related variables, and generic distress content are not clearly separated, questionnaire scores can be misread in ways that inadvertently reinforce a disease-entity narrative (“tinnitus is the problem” or “depression”) and obscure the role of modifiable processes (appraisal, attention, emotion regulation, coping/avoidance, sleep/arousal). Clearer construct language and explicit discussion of attribution wording would help prevent patients and clinicians from interpreting overlap as evidence that the percept itself is a unitary pathological condition, and instead support more accurate formulation and targeted intervention planning.

Reviewer #2: Clinically relevant and practicable paper (An ICF-based content analysis of the overlap between tinnitus and depression questionnaires).

Clinically relevant and practicable paper (An ICF-based content analysis of the overlap between tinnitus and depression questionnaires).

**********

what does this mean?). If published, this will include your full peer review and any attached files.

If you choose “no”, your identity will remain anonymous but your review may still be made public.

**Do you want your identity to be public for this peer review?**  For information about this choice, including consent withdrawal, please see our Privacy Policy

Reviewer #1: No

Reviewer #2: No

**********

[NOTE: If reviewer comments were submitted as an attachment file, they will be attached to this email and accessible via the submission site. Please log into your account, locate the manuscript record, and check for the action link "View Attachments". If this link does not appear, there are no attachment files.]

To ensure your figures meet our technical requirements, please review our figure guidelines: https://journals.plos.org/plosone/s/figures

You may also use PLOS’s free figure tool, NAAS, to help you prepare publication quality figures: https://journals.plos.org/plosone/s/figures#loc-tools-for-figure-preparation.

NAAS will assess whether your figures meet our technical requirements by comparing each figure against our figure specifications.

# Author comment: Author response to Decision Letter 1

**DOI:** 10.1371/journal.pone.0358644.r002

28 Jun 2026

Dear dr. Bojana Bukurov, reviewer #1 and reviewer #2,

We thank you for your careful consideration of our manuscript and for providing constructive feedback. We have revised our manuscript to address the points raised.

Comments from the academic editor

Regarding the comments from the Academic Editor, we have revised the manuscript to comply with PLOS ONE’s style requirements (comment 1). In addition, we have added captions for the supporting information files at the end of the manuscript and updated the corresponding in-text citations (comment 2). Comment 3 is not applicable, as the reviewers did not recommend citing any specific additional published work.

Comments from reviewer #1:

We would like to thank reviewer #1 for their detailed and insightful feedback on our manuscript. For readability, we have summarized the comments below, followed by our point-by-point responses.

1. Clarify construct validity: “depression” and “tinnitus” are heterogeneous descriptive labels, not unitary conditions

Comment: The reviewer emphasized that both depression and tinnitus are heterogeneous descriptive labels rather than unitary conditions. Regarding tinnitus specifically, the reviewer stressed the importance of distinguishing between the tinnitus percept, tinnitus-related distress as operationalized by questionnaires, and hearing loss as an audiological correlate. The reviewer recommended that findings be consistently interpreted as overlap between questionnaire operationalizations rather than between underlying conditions.

Response: We agree with this distinction and have added clarification to the introduction distinguishing between tinnitus as a self-reported percept and tinnitus-related distress as assessed by questionnaires (lines 103-107 in the clean version of the manuscript). We further specified that overlap should be interpreted at the level of questionnaire operationalization rather than as evidence of overlap between underlying conditions (lines 108-110, 353).

Regarding the point on hearing loss as an audiological correlate that is sometimes used as a proxy for tinnitus: we agree this is an important conceptual issue in the broader literature. However, as our study is confined to a content analysis of self-report questionnaires and does not involve audiological measures, this concern does not directly apply to our methodology and was therefore not further addressed in the manuscript.

2. Foreground the attribution problem: “because of tinnitus” does not measure cause and may inflate overlap by design

Comment: The reviewer noted that many tinnitus questionnaires assess generic distress domains and then add causal attribution via wording like 'because of tinnitus,' which does not measure distress caused by tinnitus in a causal sense but rather the respondent's attribution of distress to the tinnitus percept. The reviewer argued this can produce conceptual circularity and mechanically inflate overlap with depression scales, and recommended treating attribution-laden item wording as a named interpretive limitation.

Response: We have now added some information in the discussion acknowledging attribution-laden item wording in tinnitus questionnaires and its implications (lines 446-451).

3. “Body functions” dominance (Table 2) requires a more cautious interpretation

Comment: The reviewer raised concerns about the predominance of body functions in Table 2, noting that ICF places mental functions within body functions and that this should not imply physiological etiology. More specifically, the reviewer was concerned that appraisal, coping, and attention-regulation items were coded under sensory categories such as b2400, making tinnitus questionnaires appear more sensory than their content warrants. The reviewer requested explicit decision rules for these ambiguous items and a sensitivity analysis recoding them under cognitive/emotional categories.

Response: We have added a clarification in the discussion about the domain body functions (lines 358-360 & lines 512-519). Regarding the coding of items under b2400 ringing in ears or tinnitus, this was applied only to items whose core concept is inextricably linked to the tinnitus experience and cannot be separated from it, as is now also mentioned in the methods section (lines 190-201). We therefore did not perform the suggested sensitivity check, as recoding these items under mental function categories would suggest overlap with depression questionnaires where there is none, given that their content is inextricably linked to the tinnitus experience itself.

4. Methodological transparency: rater background and reliability are essential

Comment: The reviewer highlighted the importance of reporting rater backgrounds, coding procedures, and reliability measures, noting that without reliability evidence, overlap results risk being interpreted as reflecting what these particular coders decided rather than a stable property of questionnaire content.

Response: We agree with the reviewer that initiatives to make the work reproducible should be encouraged and thank them for all mentioned suggestions. Several revisions have been made to address these concerns regarding methodological transparency. We have added the rater backgrounds (lines 182-183 & 214-215) and reproducible decision rules (lines 188-212 & lines 226-232) to the method section. Furthermore, to enhance transparency we reported how many items were discussed with the third reviewer to reach consensus, including further specification of the nature of these items (lines 214-225).

5. Coarse second-level categories may overstate overlap

Comment: The reviewer raised concerns that broad second-level ICF categories such as b152 emotional functions may generate overlap even when items reflect different phenomena, such that high overlap may reflect category breadth rather than meaningful construct similarity. The reviewer suggested a sensitivity analysis using third-level categories and reporting overlap under both the current criterion and a stricter criterion incorporating item intent.

Response: We acknowledge that broad second-level ICF categories such as b152 emotional functions may generate overlap based on category breadth rather than meaningful construct similarity, which is already discussed in the limitations section (lines 501-507). At the same time, for other categories, third-level codes may create artificial distinctions between conceptually similar items, potentially leading to an underestimation of overlap. However, for readers who wish to examine the overlap at a more granular level, we have included the third-level ICF category overlap as supplementary material, which is briefly referred to in the limitations section (lines 507-511).

6. Distinguish “WHAT symptoms” from “WHY/mediating processes”

Comment: The reviewer recommended explicitly distinguishing between 'what' symptom descriptors such as low mood or sleep disturbance, and 'why' mediating processes such as cognitive appraisals, perceived control, coping strategies, and attentional biases. The reviewer argued that overlap at the 'what' level may reflect shared nonspecific distress domains, whereas overlap at the 'why' level would suggest shared interpretive and appraisal processes relevant to tinnitus distress maintenance.

Response: We agree this distinction would add valuable insight, but the ICF framework is designed to describe what people experience in terms of functioning and disability, and does not capture why or through what processes symptoms arise and are maintained. We have acknowledged this as a limitation in the discussion (lines 496-500), and consider it an interesting direction for future research.

7. Interpret the DASS finding beyond “avoid these scales”: clinical meaning of “overlap” may be relevant for emotion/distress processes, not “tinnitus”

Comment: Beyond the psychometric implications, the reviewer argued that high overlap, particularly with the DASS, may reflect that both sets of questionnaires are sampling a transdiagnostic affective distress dimension rather than two separable conditions. The reviewer encouraged discussing the clinical implications of this, suggesting that tinnitus-related suffering may be embedded in broader emotion regulation and stress vulnerability processes rather than being a tinnitus-specific symptom domain.

Response: We have expanded the discussion to include a clinical process interpretation of the overlap beyond the psychometric implications (lines 442-457).

8. Minor comments / clarifications

Comment: The reviewer requested additional justification for omitting the perspective and response option linking steps, asked for a legend to be added to Supplementary Material 2, and suggested consistently using terms such as 'tinnitus distress questionnaires' and 'depressive symptom questionnaires' to avoid reification.

Response: Regarding the minor clarifications; we have expanded on why we did not apply the perspective and response option linking rules in the methods (lines 172-177). We also added a legend to supplementary material 2 and used the terms tinnitus distress questionnaires and depressive symptom questionnaires when appropriate.

Reviewer #2:

We thank Reviewer #2 for their review of our manuscript and for highlighting its clinical relevance and practical applicability.

We appreciate the opportunity to revise our manuscript and hope that the changes and clarifications address the concerns raised. Please let us know if any further modifications are required.

Sincerely,

On behalf of all authors,

Denise Fuchten

Attachment Submitted filename: Response to reviewers.docx

# Peer review: Decision Letter 1

**DOI:** 10.1371/journal.pone.0358644.r003

**Authors:** Bojana Bukurov

7 Jul 2026

Dear Dr. Fuchten,

Thank you for submitting your manuscript to PLOS ONE. After careful consideration, we feel that it has merit but does not fully meet PLOS ONE’s publication criteria as it currently stands. Therefore, we invite you to submit a revised version of the manuscript that addresses the points raised during the review process.

Please submit your revised manuscript by Aug 21 2026 11:59PM. If you will need more time than this to complete your revisions, please reply to this message or contact the journal office at plosone@plos.org. When you're ready to submit your revision, log on to https://www.editorialmanager.com/pone/ and select the 'Submissions Needing Revision' folder to locate your manuscript file.

- A letter that responds to each point raised by the academic editor and reviewer(s). You should upload this letter as a separate file labeled 'Response to Reviewers'.
- A marked-up copy of your manuscript that highlights changes made to the original version. You should upload this as a separate file labeled 'Revised Manuscript with Track Changes'.
- An unmarked version of your revised paper without tracked changes. You should upload this as a separate file labeled 'Manuscript'.

If you would like to make changes to your financial disclosure, please include your updated statement in your cover letter. Guidelines for resubmitting your figure files are available below the reviewer comments at the end of this letter.

If applicable, we recommend that you deposit your laboratory protocols in protocols.io to enhance the reproducibility of your results. Protocols.io assigns your protocol its own identifier (DOI) so that it can be cited independently in the future. For instructions see: https://journals.plos.org/plosone/s/submission-guidelines#loc-laboratory-protocols. Additionally, PLOS ONE offers an option for publishing peer-reviewed Lab Protocol articles, which describe protocols hosted on protocols.io. Read more information on sharing protocols at https://plos.org/protocols?utm_medium=editorial-email&utm_source=authorletters&utm_campaign=protocols.

As the corresponding author, your ORCID iD is verified in the submission system and will appear in the published article. PLOS supports the use of ORCID, and we encourage all coauthors to register for an ORCID iD and use it as well. Please encourage your coauthors to verify their ORCID iD within the submission system before final acceptance, as unverified ORCID iDs will not appear in the published article. *Only*  the individual author can complete the verification step; PLOS staff *cannot*  verify ORCID iDs on behalf of authors.

We look forward to receiving your revised manuscript.

Kind regards,

Bojana Bukurov, M.D., Ph.D.

Academic Editor

PLOS One

Journal Requirements:

If the reviewer comments include a recommendation to cite specific previously published works, please review and evaluate these publications to determine whether they are relevant and should be cited. There is no requirement to cite these works unless the editor has indicated otherwise.

Please review your reference list to ensure that it is complete and correct. If you have cited papers that have been retracted, please include the rationale for doing so in the manuscript text, or remove these references and replace them with relevant current references. Any changes to the reference list should be mentioned in the rebuttal letter that accompanies your revised manuscript. If you need to cite a retracted article, indicate the article’s retracted status in the References list and also include a citation and full reference for the retraction notice.

[Note: HTML markup is below. Please do not edit.]

Reviewers' comments:

Reviewer's Responses to Questions

**Comments to the Author**

Reviewer #1: (No Response)

Reviewer #2: All comments have been addressed

**********

2. Is the manuscript technically sound, and do the data support the conclusions?

Reviewer #1: Yes

Reviewer #2: Yes

**********

3. Has the statistical analysis been performed appropriately and rigorously?

Reviewer #1: Yes

Reviewer #2: N/A

**********

4. Have the authors made all data underlying the findings in their manuscript fully available?

The PLOS Data policy

Reviewer #1: Yes

Reviewer #2: (No Response)

**********

5. Is the manuscript presented in an intelligible fashion and written in standard English?

Reviewer #1: Yes

Reviewer #2: Yes

**********

Reviewer #1: The authors' summary of my original comments is accurate, and several changes genuinely move the manuscript towards the position I was arguing for: that tinnitus, tinnitus-related distress, general distress and depression are not independent "conditions" that happen to co-occur, but overlapping operationalizations of what may be a shared, transdiagnostic affective-distress process as applied to a neutral stimulus (the tinnitus symptom) — with the ICF analysis describing questionnaire content, not disease entities. As always: Description is not explanation; and correlation is not causation.

That said, on closer inspection several of the responses are only partially implemented, and one (point 3) remains unresolved methodologically. The underlying coding decision still cuts against the very distinction the authors newly introduce in the Introduction. I go through each point below.

1. Construct validity / percept vs. distress vs. underlying condition is adequately addressed

The new paragraph (Introduction) explicitly separates the tinnitus percept from tinnitus-related distress as operationalized by questionnaires, and states that overlap should be read as overlap between instruments, not between conditions. This is the correct framing and is carried through consistently in the abstract, results, and conclusion ("questionnaires," not "tinnitus and depression," as the object of comparison).

2. Attribution problem ("because of tinnitus") is adequately addressed

The added Discussion passage (around the transdiagnostic-distress paragraph) correctly names the mechanism: generic distress items combined with causal attribution wording can inflate overlap by construction, because respondents cannot cleanly separate the source of diffuse distress from the label they attach to it. This is exactly the conceptual point I was raising, and it is now explicit rather than implicit - thanks.

3. "Body functions" dominance and the b2400 coding rule is unresolved; response appears circular

This is the one point where I don't think the revision does what it claims to. The authors added a caveat (Discussion) that "body functions" in the ICF includes mental functions and does not imply physiological etiology — fine, but that was the minor half of my comment. The substantive half was: items reflecting appraisal, coping, or attentional regulation (e.g., "how easy was it to ignore your tinnitus") are being coded under a sensory category (b2400, ringing in ears/tinnitus) rather than under attention/emotional/cognitive categories, which mechanically makes tinnitus questionnaires look more "sensory" and less "psychological" than their content warrants — and correspondingly suppresses their measured overlap with depression scales.

The authors' response to this is that such items were coded as b2400 only when their content is "inextricably linked to the tinnitus experience and cannot be separated from it," and that recoding would "suggest overlap where there is none." This is circular: whether an item like "ability to ignore tinnitus" is best understood as a description of the sensory percept or as a description of an attentional/coping process is exactly the question in dispute. Asserting inextricability is not a decision rule, it is a restatement of the conclusion. I had asked for a sensitivity analysis recoding these ambiguous items under cognitive/emotional/attentional categories; the authors declined to run it, offering only a supplementary third-level breakdown that does not address this specific ambiguity (third-level codes still nest under b2400 for these same items).

This matters for the paper's own headline finding: the TQ is reported as having by far the lowest overlap with depression measures, and the Discussion attributes this explicitly to its large share of items coded as "sensations associated with hearing and vestibular function." If some of those items are coping/appraisal items rather than percept items, the TQ's low overlap could be partly a coding artifact rather than a genuine content difference — which is precisely the kind of result inflation/deflation the paper otherwise wants to guard against (cf. their own point about the DASS potentially over-counting emotional-function items). I'd ask the authors to either run the requested sensitivity analysis or explicitly flag, as a stated limitation with the TQ result named, that the low overlap estimate is partly contingent on this coding decision.

4. Rater background and reliability is partially addressed

Rater backgrounds and the iterative decision-rule process are now well documented, and the number of items requiring third-reviewer adjudication is reported (76/262, 29%) — that is useful and appropriately transparent. However, the original protocol specified an inter-rater reliability statistic, and the authors now explicitly state this was not calculated because the iterative, discussion-based process made "a meaningful reliability statistic" undeliverable. That is a deviation from the pre-registered protocol, and while I understand the practical rationale, this leaves the central empirical claim — that overlap is a stable property of questionnaire content rather than an artifact of these two coders' judgment calls, particularly for the ~29% of items requiring adjudication — without any quantitative support, only narrative process description. At minimum, I'd ask for reporting of raw agreement before adjudication (percentage agreement, even without chance-correction, is better than nothing), since it directly bears on how much weight the overlap percentages in Table 4 can bear.

5. Coarse second-level categories is addressed

Acknowledged as a limitation, and third-level data are now provided as Supplementary Material 3 for interested readers. Given the practical constraints, reporting overlap only at the second level in the main text while providing third-level data as supplementary material is an acceptable compromise, though it does mean the headline percentages in the abstract still carry the coarse-category inflation risk without a bounded estimate alongside them.

6. "WHAT" vs. "WHY" is adequately addressed as a stated limitation

The authors correctly note this is a structural limitation of the ICF itself (a functioning/disability taxonomy, not a mechanism model) rather than something fixable within this design. Framed appropriately as a direction for future research rather than glossed over.

7. Clinical meaning of the DASS finding / transdiagnostic distress is well addressed, and the strongest revision

This is the most substantively improved part of the manuscript. The new paragraph explicitly entertains that high scores on both questionnaire types may reflect "a transdiagnostic affective distress state... not specific to either "condition" alone," with implications for how psychological tinnitus interventions are understood. This is in line with the broader empirical picture (tinnitus distress tracking general emotional-regulation and vulnerability-stress processes rather than the acoustic percept itself) and is a meaningfully different, more clinically useful statement than "avoid using the TRQ and DASS-42 together."

I'd note, though, that this framing is in some tension with point 3 above: if the authors accept that tinnitus distress may substantially reflect the same underlying affective process as depression, that is an argument FOR taking seriously the possibility that appraisal/coping items in tinnitus questionnaires are mental-function items being coded as sensory ones, not against it.

8. Minor/terminology — Mostly addressed, some cleanup still needed

The tracked-changes version still contains leftover artifacts from incomplete find-and-replace (e.g., "depression depressive symptom questionnaires," "the depressionse questionnaires," a stray "6070%" in the CES-D range) that need to be cleaned up in the final clean copy before typesetting; these look like copy-editing residue rather than substantive issues.

Summary recommendation

Points 1, 2, 6, and 7 are satisfactorily addressed and meaningfully strengthen the manuscript's conceptual framing in the direction I was advocating — treating "overlap" as a property of instruments potentially indexing a shared distress process, not as evidence that tinnitus and depression are separate comorbid illnesses. Points 5 and 8 are acceptable with minor cleanup. Points 3 and 4 are not fully resolved: the b2400 coding rule remains a load-bearing, unvalidated assumption that the paper's own headline contrast (TQ vs. TRQ) partly rests on, and the absence of any inter-rater reliability figure — even a raw pre-adjudication agreement rate — leaves the stability of the classification scheme empirically untested. I would ask for either the originally-requested sensitivity analysis (or a clearly flagged limitation naming the TQ/TFI results as coding-dependent) and a raw agreement statistic before recommending acceptance.

Reviewer #2: Clinically interesting ICF-based content analysis of the overlap between questionnaires assessing tinnitus distress and depressive symptoms.

**********

what does this mean?). If published, this will include your full peer review and any attached files.

If you choose “no”, your identity will remain anonymous but your review may still be made public.

**Do you want your identity to be public for this peer review?**  For information about this choice, including consent withdrawal, please see our Privacy Policy

Reviewer #1: No

Reviewer #2: No

**********

[NOTE: If reviewer comments were submitted as an attachment file, they will be attached to this email and accessible via the submission site. Please log into your account, locate the manuscript record, and check for the action link "View Attachments". If this link does not appear, there are no attachment files.]

To ensure your figures meet our technical requirements, please review our figure guidelines: https://journals.plos.org/plosone/s/figures

You may also use PLOS’s free figure tool, NAAS, to help you prepare publication quality figures: https://journals.plos.org/plosone/s/figures#loc-tools-for-figure-preparation.

NAAS will assess whether your figures meet our technical requirements by comparing each figure against our figure specifications.

# Author comment: Author response to Decision Letter 2

**DOI:** 10.1371/journal.pone.0358644.r004

20 Aug 2026

Dear reviewer #1,

We thank you for this careful and constructive re-review, and for confirming that several revisions move the manuscript in the intended direction. We have revised our manuscript to address the points raised, and address each of the points below following your original numbering. For brevity, quotes from the comments are excerpted to the part specifying the concern or requested action.

• Comments 1, 2, 6 and 7

Reviewer: “Points 1, 2, 6, and 7 are satisfactorily addressed and meaningfully strengthen the manuscript's conceptual framing in the direction I was advocating […].”

Response: We are happy to hear that these points are adequately addressed.

• Comment 3

Reviewer: "[…] Whether an item like 'ability to ignore tinnitus' is best understood as a description of the sensory percept or as a description of an attentional/coping process is exactly the question in dispute. […] I had asked for a sensitivity analysis recoding these ambiguous items under cognitive/emotional/attentional categories; the authors declined to run it, offering only a supplementary third-level breakdown that does not address this specific ambiguity. […] I'd ask the authors to either run the requested sensitivity analysis or explicitly flag, as a stated limitation with the TQ result named, that the low overlap estimate is partly contingent on this coding decision."

Response: Thank you for this detailed feedback. We have run the requested sensitivity analysis, and reported on this in the methods (lines 259-272 and 286-291 in the manuscript with tracked changes), results (lines 412-423) and discussion (571-585).

• Comment 4

Reviewer: “[…] At minimum, I'd ask for reporting of raw agreement before adjudication (percentage agreement, even without chance-correction, is better than nothing), since it directly bears on how much weight the overlap percentages in Table 4 can bear.”

Response: Thank you for this suggestion. We have added the requested raw agreement statistic to the result section (lines 305-313), and adjusted the method section accordingly (lines 292-299).

• Comment 5

Reviewer: “Acknowledged as a limitation, and third-level data are now provided as Supplementary Material 3 for interested readers. Given the practical constraints, reporting overlap only at the second level in the main text while providing third-level data as supplementary material is an acceptable compromise, though it does mean the headline percentages in the abstract still carry the coarse-category inflation risk without a bounded estimate alongside them.”

Response: We thank you for confirming that this is an acceptable compromise given the practical constraints. We have now added "at the second-level ICF category" next to the reported percentages in the abstract's results sentence to make the source clear to the reader.

• Comment 8

Reviewer: “The tracked-changes version still contains leftover artifacts from incomplete find-and-replace (e.g., "depression depressive symptom questionnaires," "the depressionse questionnaires," a stray "6070%" in the CES-D range) that need to be cleaned up in the final clean copy before typesetting; these look like copy-editing residue rather than substantive issues.”

Response: We thank the reviewer for flagging these. These were residual artifacts from the tracked-changes file and have been corrected in the clean copy submitted with this revision.

We appreciate the opportunity to revise our manuscript and hope that the changes and clarifications address the concerns raised. Please let us know if any further modifications are required.

Sincerely,

On behalf of all authors,

Denise Fuchten

Attachment Submitted filename: Response to reviewers 2.docx

# Peer review: Decision Letter 2

**DOI:** 10.1371/journal.pone.0358644.r005

**Authors:** Bojana Bukurov

4 Sep 2026

An ICF-based content analysis of the overlap between questionnaires assessing tinnitus distress and depressive symptoms

PONE-D-26-00978R2

Dear Dr. Denise Fuchten,

We’re pleased to inform you that your manuscript has been deemed scientifically suitable for publication and will be formally accepted once it meets all outstanding technical requirements. One of the reviewers has raised two minor issues that should be corrected before the manuscript is published. I have pasted them below for your reference.

Within one week, you’ll receive an e-mail detailing the required amendments. When these have been addressed, you’ll receive a formal acceptance letter and your manuscript will be scheduled for publication.

An invoice will be generated when your article is formally accepted. Please note, if your institution has a publishing partnership with PLOS and your article meets the relevant criteria, all or part of your publication costs will be covered. Please make sure your user information is up-to-date by logging into Editorial Manager at Editorial Manager® and clicking the ‘Update My Information' link at the top of the page. For questions related to billing, please contact billing support.

If your institution or institutions have a press office, please notify them about your upcoming paper to help maximize its impact. If they’ll be preparing press materials, please inform our press team as soon as possible -- no later than 48 hours after receiving the formal acceptance. Your manuscript will remain under strict press embargo until 2 pm Eastern Time on the date of publication. For more information, please contact onepress@plos.org.

Kind regards,

Bojana Bukurov, M.D., Ph.D.

Academic Editor

PLOS One

(Reviewers' comments:

Reviewer's Responses to Questions

**Comments to the Author**

Reviewer #1: All comments have been addressed

Reviewer #2: All comments have been addressed

**********

2. Is the manuscript technically sound, and do the data support the conclusions?

Reviewer #1: Yes

Reviewer #2: Yes

**********

3. Has the statistical analysis been performed appropriately and rigorously?

Reviewer #1: Yes

Reviewer #2: Yes

**********

4. Have the authors made all data underlying the findings in their manuscript fully available?

The PLOS Data policy

Reviewer #1: Yes

Reviewer #2: Yes

**********

5. Is the manuscript presented in an intelligible fashion and written in standard English?

Reviewer #1: Yes

Reviewer #2: Yes

**********

Reviewer #1: Thank you for addressing my previous comments. The sensitivity analysis of the b240 items is a useful addition, and my earlier concerns are now resolved.

I have only a few minor terminological points, which can be handled at the editorial stage:

(1) Tinnitus and depression are referred to as “conditions” at several points (clean copy ll. 84, 476, 497, 570, 580). I suggest replacing this with “the two symptom domains” throughout. For tinnitus, this is important given the distinction made in the Introduction (ll. 69–74) between tinnitus as a stimulus and tinnitus disorder as the associated distress.

Likewise, depression is not a condition in any straightforward sense, despite the common usage of the term as such. There is substantial evidence that the depressive syndrome is heterogeneous and ill-defined, with important implications for research progress. Here, the object of study is depressive symptomatology as operationalised by the questionnaires (ll. 107–108), rather than a unitary condition. At l. 570, for example, “does not imply that tinnitus distress or depressive symptoms are primarily physiological in origin” would be preferable.It may also be worth noting that the wide range of overlap across the depression questionnaires (40–60% for the SDS vs. 85.71–100% for the DASS-42) is consistent with this heterogeneity.

(2) The statement at ll. 111–114 regarding partially overlapping and sometimes opposing dimensions of depression should have a reference. Fried & Nesse (2015) and Lynall & McIntosh (2023) would be appropriate citations:

Fried, E. I., & Nesse, R. M. (2015). Depression is not a consistent syndrome: An investigation of unique symptom patterns in the STAR*D study. Journal of Affective Disorders, 172, 96–102.

Lynall, M. E., & McIntosh, A. M. (2023). The heterogeneity of depression. American Journal of Psychiatry, 180(10), 703–704.

Subject to these minor changes, I am happy to recommend publication. I do not need to see the manuscript again.

Reviewer #2: A clinically relevant ICF-based content analysis of the overlap between questionnaires assessing tinnitus distress and depressive symptoms.

**********

what does this mean?). If published, this will include your full peer review and any attached files.

If you choose “no”, your identity will remain anonymous but your review may still be made public.

**Do you want your identity to be public for this peer review?**  For information about this choice, including consent withdrawal, please see our Privacy Policy

Reviewer #1: No

Reviewer #2: No

**********

# Editor report: Acceptance letter

**DOI:** 10.1371/journal.pone.0358644.r006

**Authors:** Bojana Bukurov

PONE-D-26-00978R2

PLOS One

Dear Dr. Fuchten,

I'm pleased to inform you that your manuscript has been deemed suitable for publication in PLOS One. Congratulations! Your manuscript is now being handed over to our production team.

At this stage, our production department will prepare your paper for publication. This includes ensuring the following:

* All references, tables, and figures are properly cited

* All relevant supporting information is included in the manuscript submission,

* There are no issues that prevent the paper from being properly typeset

You will receive further instructions from the production team, including instructions on how to review your proof when it is ready. Please keep in mind that we are working through a large volume of accepted articles, so please give us a few days to review your paper and let you know the next and final steps.

Lastly, if your institution or institutions have a press office, please let them know about your upcoming paper now to help maximize its impact. If they'll be preparing press materials, please inform our press team within the next 48 hours. Your manuscript will remain under strict press embargo until 2 pm Eastern Time on the date of publication. For more information, please contact onepress@plos.org.

You will receive an invoice from PLOS for your publication fee after your manuscript has reached the completed accept phase. If you receive an email requesting payment before acceptance or for any other service, this may be a phishing scheme. Learn how to identify phishing emails and protect your accounts at https://explore.plos.org/phishing.

If we can help with anything else, please email us at customercare@plos.org.

Thank you for submitting your work to PLOS One and supporting open access.

Kind regards,

PLOS One Editorial Office Staff

on behalf of

Ass. prof. Bojana Bukurov

Academic Editor

PLOS One
